# Biochanin A, a Plant Isoflavone, Disrupts Peptidoglycan Biosynthesis by Downregulating *femA* and *femB*, and Impairs Cell Wall Integrity in Multidrug-Resistant *Staphylococcus aureus*

**DOI:** 10.3390/antibiotics15020195

**Published:** 2026-02-10

**Authors:** Jade Joshua R. Teodosio, Kathryn Ann H. Dizon, Julyanna R. Bruna, Jan Vincent N. Sollesta, Zenith M. Villorente, Jonel P. Saludes, Doralyn S. Dalisay

**Affiliations:** 1 Center for Chemical Biology and Biotechnology (C2B2), University of San Agustin, Iloilo City 5000, Philippines; jjteodosio@usa.edu.ph (J.J.R.T.); kadizon@usa.edu.ph (K.A.H.D.); julyanna@usa.edu.ph (J.R.B.); 2 Maridan Industries, Inc., Iloilo City 5000, Philippines; jvsollesta@maridan.com.ph (J.V.N.S.); zmvillorente@maridan.com.ph (Z.M.V.); 3 Center for Natural Drug Discovery and Development (CND3), University of San Agustin, Iloilo City 5000, Philippines; jsaludes@usa.edu.ph; 4 Department of Chemistry, University of San Agustin, Iloilo City 5000, Philippines; 5 Balik Scientist Program, Department of Science and Technology-Philippine Council for Health Research and Development, Taguig City 1631, Philippines; 6 Department of Biology, University of San Agustin, Iloilo City 5000, Philippines

**Keywords:** antibiotic, isoflavone, biochanin A, *femA*, *femB*, MDR-SA, peptidoglycan, mechanism-of-action

## Abstract

**Background/Objectives**: The global rise in multidrug-resistant *Staphylococcus aureus* (MDR-SA) threatens the efficacy of existing antibiotics and necessitates alternative antibacterial strategies. Plant-derived isoflavones represent a promising but underexplored source of novel antimicrobials. Biochanin A, isolated from *Cajanus cajan* seeds, exhibits antibacterial activity and may act via noncanonical mechanisms. This study elucidates the mechanism of action and safety profile of Biochanin A against MDR-SA using integrated experimental and computational approaches. **Methods**: Antibacterial activity was assessed by minimum inhibitory concentration (MIC) testing. Membrane integrity and morphological alterations were evaluated using flow cytometry and scanning electron microscopy (SEM), respectively. Target gene modulation was analyzed by qRT-PCR, while molecular interactions were examined through in silico docking. Cytotoxicity was evaluated in normal mammalian kidney, liver, and cardiac cells. **Results**: Biochanin A inhibited MDR-SA with an MIC80 of 64 µg/mL. Flow cytometry showed membrane disruption in 74.46 ± 13.19% of treated cells, and SEM revealed a 20% reduction in cell size (561.95 ± 21.99 nm). Biochanin A markedly downregulated *femA* (94%) and *femB* (67%), with minimal effect on *femX* (10%). Docking analyses supported preferential binding to FemA (−7.7 kcal/mol) and FemB (−7.5 kcal/mol) proteins. No cytotoxic effects were observed in normal mammalian cells. **Conclusions**: Biochanin A is a promising plant-derived antibacterial candidate against MDR-SA, targeting key cell wall biosynthesis genes while maintaining mammalian safety. These findings position Biochanin A as a viable lead for further biochemical, structural, and in vivo pharmacological validation, highlighting the translational potential of plant-derived isoflavones in combating antibiotic resistance.

## 1. Introduction

Multidrug-resistant *Staphylococcus aureus* (MDR-SA) continues to exert significant pressure on healthcare systems [1] and the global economy [2]. Owing to the increasing prevalence of resistance to multiple antibiotic classes, mortality from staphylococcal infections has reached up to 1 million deaths annually, accounting for approximately 20% of global antimicrobial resistance (AMR)-related fatalities [3]. The high adaptability of MDR-SA presents major challenges to conventional therapeutic strategies. Resistance has been reported against numerous antibiotic classes, including beta-lactams [4], cephalosporins [5], carbapenems [6], tetracyclines [7], glycopeptides [8], lipoglycopeptides [9], phosphonic acids [10], lipopeptides [11], oxazolidinones [12], macrolides [13], chloramphenicol [14], and aminoglycosides [15]. This alarming trend has severely compromised the global antibiotic pipeline, prompting the World Health Organization (WHO) to classify *S. aureus*, including MDR-SA, as a High Priority Pathogen, underscoring the urgent need for novel antibacterial agents [16].

Bacterial peptidoglycan (PG) is composed of three principal components: disaccharides, stem peptides, and interpeptide bridge segments. The conserved disaccharide backbone consists of *N*-acetylglucosamine and *N*-acetylmuramic acid [17], whereas stem peptides and bridge structures vary among bacterial species. In MDR-SA, the stem peptide comprises a pentapeptide sequence of L-alanine, D-isoglutamine, L-lysine, and two D-alanine residues, while the interpeptide bridge contains a characteristic pentaglycine structure [18]. Final PG assembly requires the coordinated action of transglycosylase and transpeptidase enzymes at the outer surface of the cytoplasmic membrane. Transglycosylase catalyzes the polymerization of glycan chains via beta-glycosidic bond formation, while transpeptidase cross-links adjacent chains to generate a rigid three-dimensional PG lattice that provides structural integrity to the bacterial cell wall [19].

Pentaglycine interpeptide bridge biosynthesis in *S. aureus* is a highly ordered, multistep process mediated sequentially by the enzymes FemX, FemA, and FemB [20,21,22,23,24]. FemX (lipid II:glycine glycyltransferase), encoded by *femX* gene, catalyzes the transfer of the first glycine residue to the epsilon-amino group of L-lysine at position three of the lipid II-linked pentapeptide [22]. FemA, encoded by *femA* gene, subsequently adds two glycine residues to form a triglycine side chain [23,24]. On the other hand, FemB, encoded by *femB* gene, completes the process by appending the fourth and fifth glycine residues, yielding the mature pentaglycine interpeptide bridge [24,25]. This structure is critical for efficient cross-linking of peptidoglycan strands, and disruption of any step in the FemX-FemA-FemB cascade may compromise cell wall integrity and bacterial viability [25,26]. Consequently, these enzymes represent attractive targets for anti-staphylococcal drug development [27,28].

Plant-derived compounds have gained increasing attention as sources of structurally diverse and biologically active antibacterial agents [29]. Among these, flavonoids are particularly promising due to their target-specific and broad-spectrum antibacterial activities against both Gram-positive and Gram-negative pathogens [29,30]. Within this class, Biochanin A has emerged as a potential antibacterial candidate against MDR-SA [29,30,31,32,33,34]. In our previous work, Biochanin A isolated from *Cajanus cajan* seeds demonstrated notable antibacterial activity against MDR-SA and other *S. aureus* strains [35]. In addition, Biochanin A has been extensively studied for its diverse pharmacological activities, particularly its anticancer effects against lung [36], prostate [37], gastrointestinal [38], breast [39], and osteosarcoma [40]. These activities are attributed to inhibition of cytochrome P450-mediated carcinogen metabolism, suppression of COX-2-driven inflammatory pathways, enhancement of radiotherapy efficacy with minimal toxicity to normal tissues, and modulation of oncogenic signaling cascades [41]. Meanwhile, Biochanin A exhibits antiviral activity against human herpesvirus 6 and influenza A by disrupting host signaling pathways essential for viral replication [42].

However, the antibacterial potential of Biochanin A remains relatively underexplored, partly due to solubility limitations at higher concentrations [43]. Existing studies predominantly emphasize its synergistic effects with conventional antibiotics rather than its independent antibacterial activity [44,45,46,47,48]. Nevertheless, Biochanin A has demonstrated inhibitory activity against *Clostridia* [45], *Pseudomonas aeruginosa* [45], *Escherichia coli* [46], *Chlamydia pneumoniae* [47], and *Xanthomonas axonopodis* [48]. Despite these findings, systematic investigations into Biochanin A’s cytotoxicity and its antibacterial mechanism of action against MDR-SA remain limited.

To address this gap, this study investigates the antibacterial mechanisms of Biochanin A against MDR-SA using a multimodal approach. Flow cytometry and scanning electron microscopy were employed to assess membrane integrity and morphological alterations, while relative gene expression analysis via qRT-PCR targeted key peptidoglycan biosynthesis genes, including *femX*, *femA*, and *femB*. Molecular docking was conducted to examine interactions between Biochanin A and the corresponding Fem proteins (FemX, FemA, and FemB). In parallel, lactate dehydrogenase-based cytotoxicity assays evaluated the safety of Biochanin A in kidney, liver, and cardiac cell models. Collectively, these findings provide mechanistic insight into how Biochanin A disrupts cell wall integrity and supports its potential as a plant-derived antibacterial agent against MDR-SA.

## 2. Results

### 2.1. Minimum Inhibitory Concentration (MIC)

**Figure 1:**
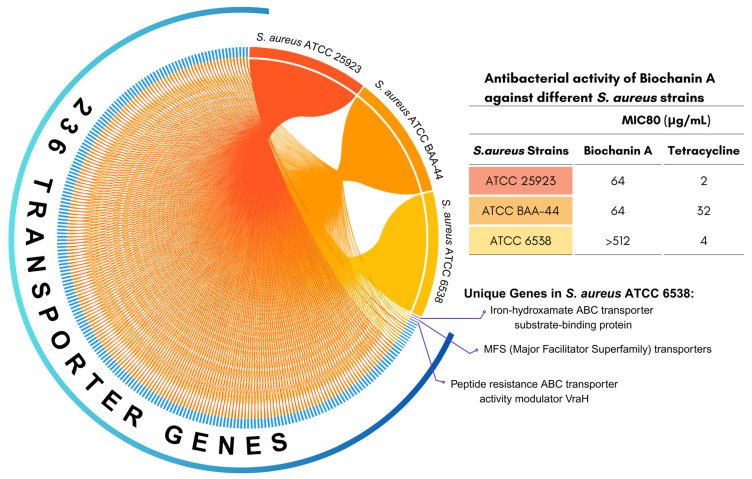
Antibacterial activity of Biochanin A against three ATCC *S. aureus* strains (upper right). Chord diagram in Circos Table Viewer illustrating a pan-genome comparative analysis of transporter genes (blue) present in *S. aureus* ATCC 25923 (red), *S. aureus* ATCC BAA-44 (orange), and *S. aureus* ATCC 6538 (yellow). Three unique genes (purple) were annotated as present only in *S. aureus* ATCC 6538.

To determine Biochanin A’s antibacterial activity against various strains of *S. aureus*, its minimum inhibitory concentration (MIC) was assessed against three *S. aureus* strains: ATCC BAA-44 (MDR strain) and non-resistant strains ATCC 25923 and ATCC 6xd8 was used in this study. The microbial growth inhibition > 80% was considered as the MIC value and annotated as MIC80. The results indicated that Biochanin A displayed antibacterial activity against *S. aureus* ATCC BAA-44 and *S. aureus* ATCC 25923, with both showing an MIC80 of 64 µg/mL (Figure 1). However, Biochanin A showed MIC80 value of > 512 µg/mL when tested against *S. aureus* ATCC 6538, indicating that it is unlikely to inhibit the growth of this specific strain at low concentrations.

### 2.2. Comparative Genome Analysis on Genes Encoding Membrane-Associated Transport Proteins

To investigate the differential susceptibility of *S. aureus* strains to Biochanin A, we hypothesized that resistance in *S. aureus* ATCC 6538 may be attributable to an enhanced transport system that restricts intracellular accumulation of Biochanin A. Reduced intracellular drug accumulation, primarily mediated by active efflux, is a well-established mechanism of bacterial resistance [49,50,51]. To evaluate this, we conducted a comparative in silico genomic analysis with a targeted focus on genes encoding membrane-associated transport proteins. These proteins are known to play a critical role in bacterial defense mechanisms by mediating the active efflux of antimicrobial agents, thereby decreasing their intracellular concentration and therapeutic efficacy [49,50,51]. It was revealed that *S. aureus* ATCC 6538 possesses 236 transporter genes, making it the strain with the highest number of transporter genes in the group. Interestingly, three of these transporter genes are unique to *S. aureus* ATCC 6538 and are absent in both *S. aureus* ATCC BAA-44 and *S. aureus* ATCC 25923. These unique genes include the iron-hydroxamate ABC transporter substrate-binding protein, major facilitator superfamily (MFS) transporter, and the peptide resistance ABC transporter activity modulator *VraH* (Figure 1). These genes encode membrane-associated proteins involved in the selective transport of substrates across the bacterial membrane, mediating the efflux and influx of compounds [49,50,51]. In comparison, *S. aureus* ATCC BAA-44 harbors 231 transporter genes, while *S. aureus* ATCC 25923 contains 225 transporter genes (Table S1). This variation in the composition of transporter genes among the three *S. aureus* strains may contribute to the strain-specific differences in Biochanin A susceptibility and partially explain the observed resistance in *S. aureus* ATCC 6538.

### 2.3. Bacterial Cell Viability Using Spread Plate Method

**Figure 2:**
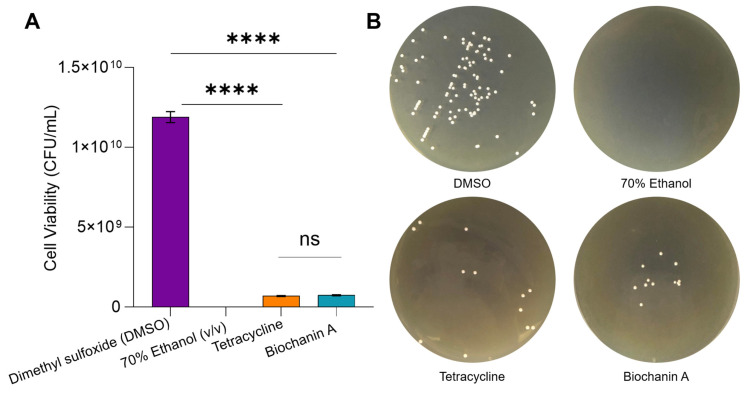
Effect of Biochanin A on the viability of *S. aureus* ATCC BAA-44. (**A**) Statistical analysis of bacterial viability of MDR-SA after exposure to the negative control dimethyl sulfoxide (DMSO), 64 µg/mL tetracycline, and 70% Ethanol (*v*/*v*) as the positive control, as well as 64 µg/mL Biochanin A, was conducted using one-way ANOVA through GraphPad Prism v.9.3.1. The error bars represent the standard error of the mean (±SEM). Statistical analysis is indicated by (ns) in the bar graph, signifying no significant difference between tested samples (*p* > 0.05), while (****) denotes significance (*p* < 0.05). (**B**) Spread plate assay illustrating the bacteriostatic effect of the negative control, DMSO, the positive controls 70% Ethanol (*v*/*v*) and 64 µg/mL tetracycline, and 64 µg/mL Biochanin A on *S. aureus* ATCC BAA-44 following treatment.

Since Biochanin A exhibited an MIC80 of 64 µg/mL against *S. aureus* ATCC BAA-44, this multidrug-resistant strain was selected for further study. Its resistance to multiple antibiotics and the limited reports describing the mechanism of action of Biochanin A against this strain underscore its relevance for mechanistic investigation. The results indicated that Biochanin A induces a bacteriostatic effect after 24 h exposure suggesting that bacteria could still grow after Biochanin A treatment. Nonetheless, Biochanin A significantly reduced the viable colonies of *S. aureus* ATCC BAA-44 by 90%. The colony-forming units (CFU) of MDR-SA in the Biochanin A-treated plates were 7.39 × 10^8^ ± 3.61 × 10^7^ CFU/mL, which was significantly lower compared to the control 2.5% (*v*/*v*) dimethyl sulfoxide (DMSO), which exhibited a viable count of 1.19 × 10^10^ ± 3.43 × 10^7^ CFU/mL. Additionally, the effect of Biochanin A in reducing the viability of MDR-SA at its MIC80 concentration showed no significant difference (*p* > 0.05) when compared to tetracycline, a known antibiotic standard for MDR-SA, which showed a viable count of 6.89 × 10^8^ ± 3.43 × 10^7^ CFU/mL (Figure 2).

### 2.4. Propidium Iodide and Calcein Uptake Assay Using Flow Cytometer

**Figure 3:**
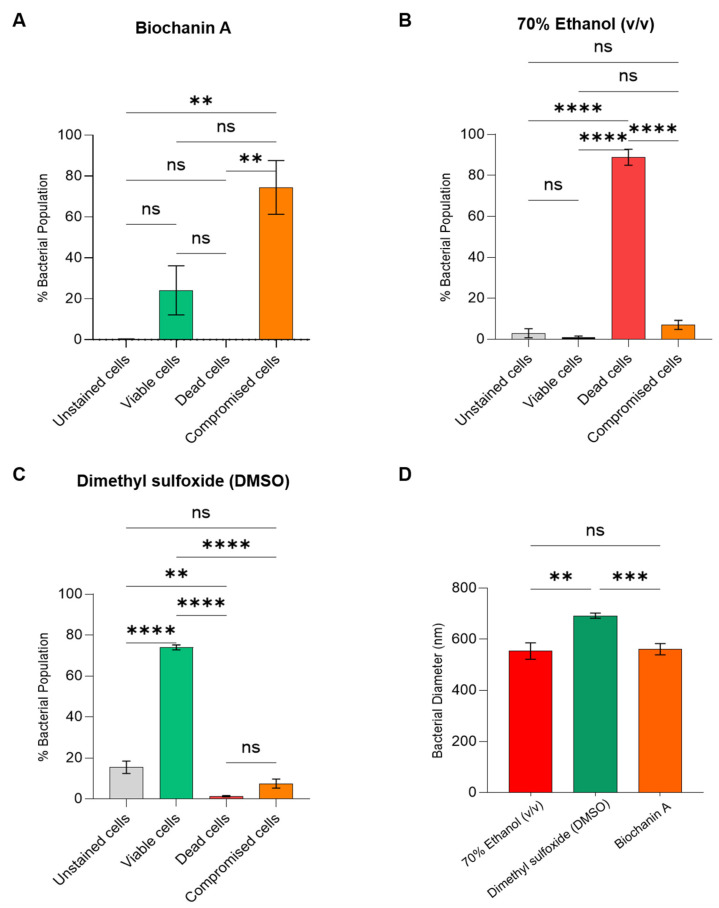
The statistical analysis of the live/dead ratio was conducted using flow cytometry in the bacterial population of MDR-SA treated with (**A**) 64 µg/mL Biochanin A, (**B**) 70% Ethanol (*v*/*v*) as the positive control, and (**C**) DMSO as negative control. (**D**) The bacterial diameter of MDR-SA after exposure to 70% Ethanol (*v*/*v*) as the positive control (red), DMSO as the negative control (green), and Biochanin A at 64 µg/mL (orange). The error bars in the graph represent ±SEM. One-way ANOVA paired with Tukey’s test was used to determine the statistical difference between treatments, wherein there is no significance (ns) if the *p*-value is > 0.05, and significance in the results (**), (***), and (****) if the *p*-value is < 0.05.

**Figure 4:**
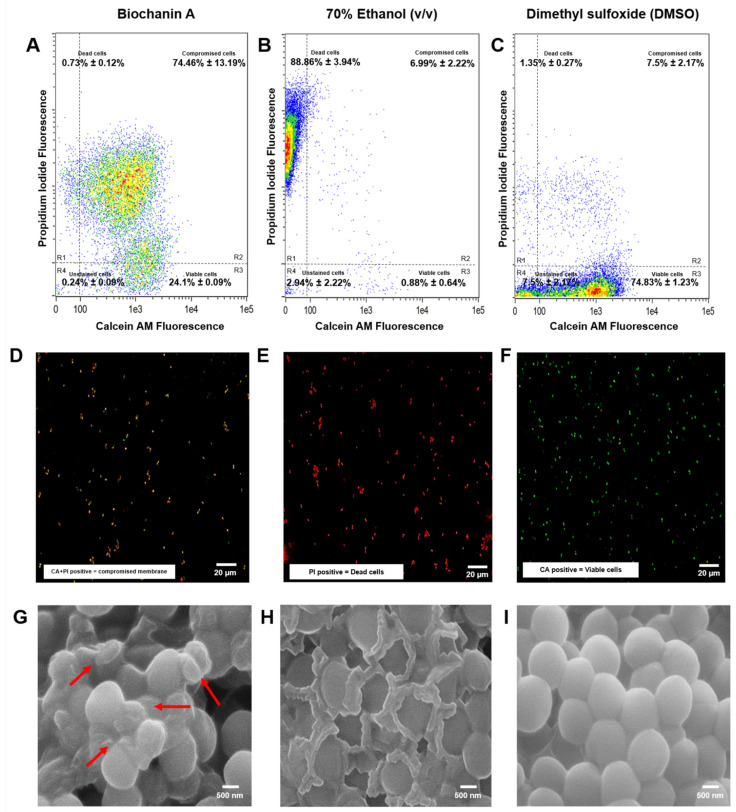
Evaluation of *S. aureus* ATCC BAA-44 viability and morphology following treatment. (**A**–**C**) Flow cytometry analysis of bacterial populations stained with Calcein-AM (CA) and propidium iodide (PI). (**A**) Treatment with 64 µg/mL Biochanin A demonstrates a shift towards R1 and R2, indicating significant membrane disruption and partial loss of viability. (**B**) Treatment with 70% Ethanol (*v*/*v*) as a positive control result in a predominant clustering in R1 (strong PI signal), indicating a high proportion of dead cells. (**C**) Treatment with DMSO as a negative control primarily shows clustering in R3 (strong CA signal), representing viable cells with intact. (**D**–**F**) Fluorescence microscopy visualization of stained cells. (**D**) 64 µg/mL Biochanin A -treated cells show a combination of red and green fluorescence, reflecting partial membrane disruption and loss of cell viability. (**E**) 70% Ethanol (*v*/*v*)-treated cells exhibit red fluorescence due to PI uptake, signifying extensive membrane damage and cell death. (**F**) DMSO-treated cells display green fluorescence from CA, indicative of intact membranes and metabolic activity. (**G**–**I**) Scanning electron microscopy (SEM) imaging of bacterial morphology. (**G**) Biochanin A-treated cells at 64 µg/mL reveal surface disruptions and altered shapes (red arrows), confirming the compound’s effect on cell morphology. (**H**) Cells treated with 70% Ethanol (*v*/*v*) exhibit collapsed structures and irregular shapes, indicating severe structural damage. (**I**) DMSO-treated cells have smooth surfaces and normal morphology, consistent with unaffected bacterial integrity.

The live/dead assay results indicated that 0.73 ± 0.12% of the *S. aureus* ATCC BAA-44 population treated with Biochanin A at 64 µg/mL was detected in R1 (Figure 3A and Figure 4A), corresponding to dead bacterial cells. This percentage of dead cells is significantly lower than the R1 percentage for the positive control, 70% Ethanol (*v*/*v*), which showed 88.86 ± 3.94% (Figure 3B and Figure 4B). In contrast, Biochanin A demonstrated a lower percentage of viable cells, with 24.1 ± 12.41% detected in R3 compared to the negative control, DMSO, in the same region (Figure 3C and Figure 4C). Notably, most of the *S. aureus* ATCC BAA-44 population treated with Biochanin A at 64 µg/mL exhibited compromised membrane integrity after exposure, as observed in R2, where 74.46 ± 13.19% of the MDR-SA population fluoresced in the presence of both PI and calcein dyes (Figure 3A and Figure 4A).

### 2.5. MDR-SA Bacterial Membrane Is Compromised upon Treatment with Biochanin A as Indicated in Fluorescence Microscopy Analysis

The physiological function of the MDR-SA bacterial membrane was evaluated by assessing its esterase activity with fluorescent probes, including calcein AM (CA) and propidium iodide (PI). In this experiment, MDR-SA cells treated with MIC80 concentration (64 µg/mL) Biochanin A displayed double-stained fluorescence (Figure 4D) compared to positive control 70% Ethanol (*v*/*v*)-treated MDR-SA population, which showed strong PI red fluorescence (Figure 4E) indicating dead cells. Meanwhile, the negative control DMSO exhibited high green fluorescence due to the presence of CA (Figure 4F).

### 2.6. Scanning Electron Microscopy (SEM) Reveals Morphological Effects of Biochanin A on MDR-SA Cells

The effects of Biochanin A on the morphology of MDR-SA were assessed using scanning electron microscopy (SEM). MDR-SA cells treated with Biochanin A at 64 µg/mL exhibited significant morphological collapse, evidenced by a 20% reduction in size to 561.95 ± 21.99 nm (Figure 4G). A distinct burst-like structure was observed in MDR-SA cells treated with 70% Ethanol (*v*/*v*), resulting in a size reduction to 553.63 ± 31.72 nm (Figure 4H). In contrast, untreated cells exposed to the negative control DMSO retained their characteristic intact spherical morphology, averaging 692.03 ± 10.85 nm in size (Figure 4I). Statistical analysis using one-way ANOVA revealed that the effect of Biochanin A in reducing the bacterial morphology size of MDR-SA showed no significant difference, with a *p*-value of > 0.05 (Figure 3D) when compared to the positive control 70% Ethanol (*v*/*v*).

### 2.7. Relative Gene Expression Using Quantitative Real-Time Polymerase Chain Reaction (qRT-PCR)

**Figure 5:**
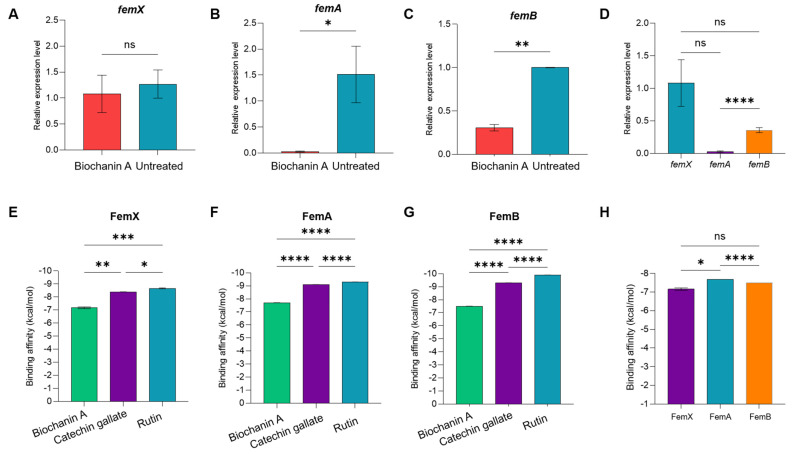
Gene expression analysis of (**A**) *femX*, (**B**) *femA*, and (**C**) *femB* in *S. aureus* ATCC BAA-44 treated with 64 µg/mL Biochanin A. (**D**) Comparison of expression levels of *femX* (blue), *femA* (violet), and *femB* (orange) after treatment with 64 µg/mL Biochanin A. Molecular docking analysis of Biochanin A (green), control compounds catechin gallate (violet) and rutin (blue) against (**E**) FemX, (**F**) FemA, and (**G**) FemB. (**H**) Summary of binding affinity of Biochanin A against FemX (violet), FemA (blue), and FemB (orange). Error bars represent ±SEM, while (*) and (**), (***), and (****) denote *p* < 0.05.

To further elucidate the mechanism of action of Biochanin A against MDR-SA at the molecular level, we analyzed the expression levels of key genes involved in PG synthesis, namely *femX*, *femA*, and *femB*, using qRT-PCR for relative gene expression analysis. These genes are essential for PG biosynthesis [21], a crucial component of the bacterial cell wall that serves as structural barrier and a defense mechanism against antibiotics, including β-lactams [52]. Gene expression analysis via qRT-PCR revealed downregulation of *femA* and *femB* expression in MDR-SA cells treated with MIC80 concentration (64 µg/mL) of Biochanin A. A minimal reduction (10% decrease) in *femX* expression level was observed with Biochanin A treatment (1.08 ± 0.35-fold) compared to the untreated control (1.2 ± 0.27-fold), but this was not statistically significant (*p* > 0.05) (Figure 5A). Moreover, *femA* expression significantly decreased to 0.028 ± 0.01-fold, representing approximately a 94% reduction compared to the untreated control (1.51 ± 0.54-fold) (Figure 5B). Meanwhile, *femB* expression moderately decreased to 0.35 ± 0.039-fold (Figure 5C), indicating a 67% reduction compared to the untreated control showing 1.06 ± 0.15-fold. The downregulation of both *femA* and *femB* gene expression in Biochanin A-treated groups was significant compared to their respective untreated controls, according to statistical analysis using an unpaired *t*-test with Welch’s correction, where *p* < 0.05. Conversely, Biochanin A significantly reduced the expression level of *femA* compared to *femX* and *femB*, as revealed by one-way ANOVA where *p* < 0.05 (Figure 5D), suggesting that Biochanin A is more effective in downregulating *femA* expression than *femB* without targeting *femX*.

### 2.8. In Silico Analyses Showing Biochanin A’s Interaction with FemX, FemA, and FemB Proteins

**Figure 6:**
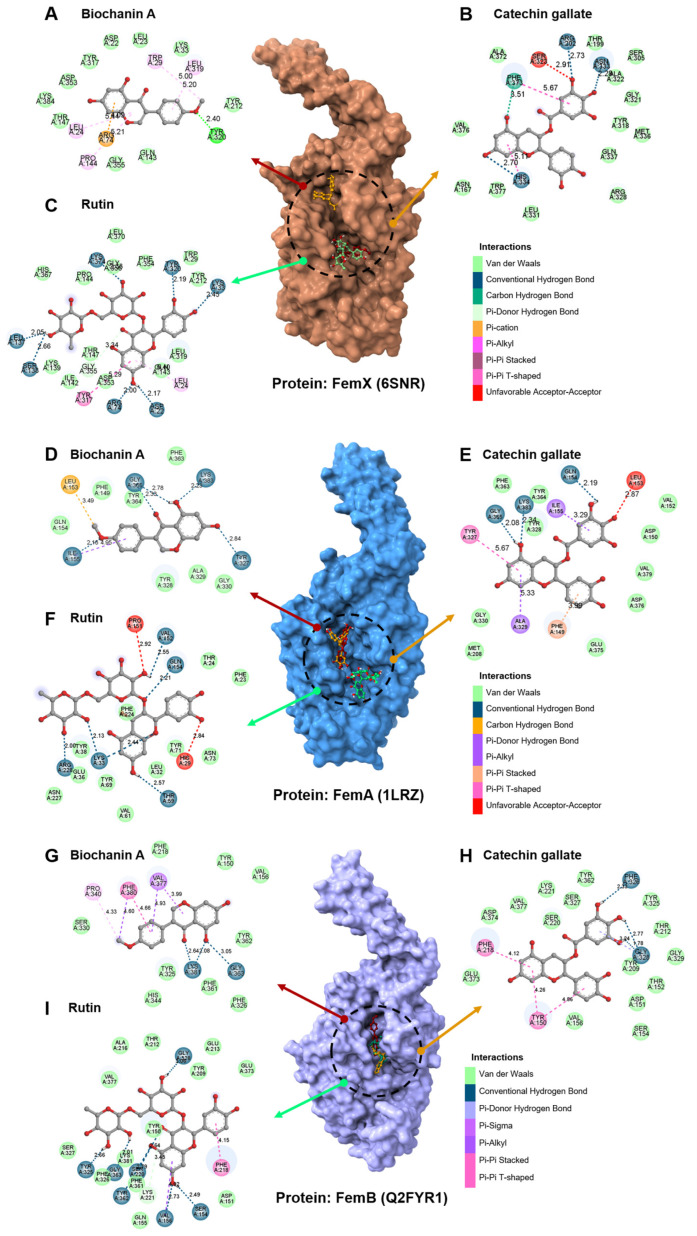
Two-dimensional illustration of binding interaction of (**A**,**D**,**G**) Biochanin A; (**B**,**E**,**H**) catechin gallate; and (**C**,**F**,**I**) rutin against FemX, FemA, and FemB displayed in 3D.

In silico analyses were performed to further elucidate Biochanin A’s molecular mechanism of action against MDR-SA. Molecular docking demonstrated that Biochanin A shows binding affinities of −7.1 kcal/mol, −7.7 kcal/mol, and −7.5 kcal/mol for FemX (Figure 5E), FemA (Figure 5F), and FemB (Figure 5G), respectively. Within the active site of FemX, a π-cation interaction with ARG74 was identified. A π-alkyl interaction was noted in LEU24, PRO144, LEU319, TRP29, along with van der Waals interactions in GLY355, GLN143, TYR212, LYS33, LEU23, ASP22, TYR317, ASP353, LYS384, and THR147. Hydrogen bonding was observed only with TYR320, indicating that Biochanin A exhibits weak interactions with FemX protein (Figure 6A). Furthermore, Biochanin A formed hydrogen bonds with critical amino acid residues of FemA protein, including LEU153, ILE155, TYR327, GLY365, and LYS383. Van der Waals interactions were also detected with PHE149, GLN154, TYR328, ALA329, GLY330, PHE363, and TYR364 (Figure 6D).

Meanwhile, Biochanin A interacted with FemB protein by forming hydrogen bonds with LYS381, GLY363, VAL377, and PHE380. It also established π-π interactions with PHE380, PRO340, and VAL377. Furthermore, van der Waals interactions were observed with PHE218, TYR150, VAL156, SER330, TYR325, HIS344, and TYR362 (Figure 6G) (Table S3). Control compounds catechin gallate (−8.4, −9.1 and −9.3 kcal/mol) (Figure 6B,E,H) and rutin (−8.6, −8.7, and −9.9 kcal/mol) (Figure 6C,F,I) demonstrated stronger binding affinities than Biochanin A against FemX, FemA, and FemB, despite Biochanin A’s inferior binding affinities towards the target proteins compared to those of its flavonoid counterparts. Conversely, Biochanin A does not violate Lipinski’s rule of five compared to catechin gallate and rutin. It shows superior absorption and bioavailability, as revealed in silico pharmacokinetic study using pkCSM (Table S4). Biochanin A has the highest Caco-2 permeability (0.897 log Papp 10^6^) and human intestinal absorption (93.03%), compared to catechin gallate (62.10%) and rutin (23.45%). Although its water solubility was lower (−3.735 log mol/L), this can be improved with advanced drug formulations (Table S4). Additionally, while all three compounds are substrates for P-glycoproteins, Biochanin A’s lack of inhibition of P-glycoprotein I and II minimizes drug-drug interactions and enhances systemic bioavailability. Biochanin A also exhibited the lowest steady-state volume of distribution (VDss: −0.341 log L/kg), indicating that it is primarily confined to plasma rather than spreading extensively into tissues (Table S4). This reduces off-target effects and enhances therapeutic focus. Its low fraction unbound (Fu: 0.03) supports a longer plasma half-life and sustained therapeutic effects despite strong plasma protein binding. Furthermore, Biochanin A showed better blood-brain barrier permeability (log BBB: −0.221) and CNS permeability (log PS: −2.115) than catechin gallate and rutin, suggesting potential central nervous system activity (Table S4).

Metabolically, Biochanin A serves as a substrate for CYP3A4, a key enzyme in drug metabolism. Additionally, Biochanin A inhibits CYP1A2, CYP2C19, and CYP2C9, which may facilitate pathway-specific modulation. In contrast, catechin gallate and rutin exhibit minimal interaction with metabolic enzymes, potentially reducing their systemic activation and efficacy (Table S4). Biochanin A demonstrated the highest total clearance rate (0.247 log mL/min/kg), indicating efficient elimination and a lower risk of drug accumulation. Although this may shorten its half-life, it aids in minimizing toxicity risks. Furthermore, none of the compounds, including Biochanin A, are substrates for the renal OCT2 transporter, thereby decreasing the likelihood of renal-related side effects (Table S4). The safety profile of Biochanin A further bolsters its potential as a drug candidate, as it was found non-mutagenic (negative AMES test), non-hepatotoxic, and free from the risks of skin sensitization. It also displayed moderate oral acute toxicity (LD50: 1.851 mol/kg) and a manageable chronic toxicity threshold (LOAEL: 1.142 log mg/kg/bw/day). While catechin gallate and rutin showed slightly higher tolerated doses, their poor absorption and distribution profiles limit their therapeutic potential compared to Biochanin A (Table S4).

### 2.9. Lactate Dehydrogenase (LDH) Toxicity Assay

**Figure 7:**
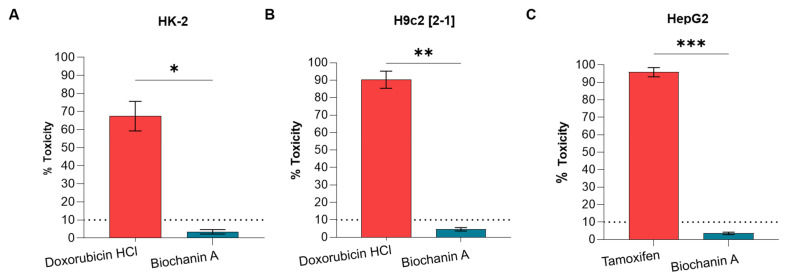
Toxicity profile of Biochanin A against (**A**) kidney (HK-2), (**B**) cardiac (H9c2[2-1]), and (**C**) liver (HepG2) cells. The positive controls used were doxorubicin hydrochloride at concentrations of 108.7 µg/mL and 92.7 µg/mL for the kidney (HK-2) and cardiac (H9c2[2-1]) cell lines, respectively, and tamoxifen for the liver carcinoma cell line (HepG2) at a concentration of 14.9 µg/mL. The error bars indicate the standard error of the mean ± SEM across three trials. The error bars represent the ±SEM, while (*), (**), and (***) denote *p* < 0.05. The dotted line in the bar graph indicates ten percent toxicity threshold as acceptable cytoxicity range.

To establish the safety of Biochanin A in normal and model cell lines, we utilized kidney HK-2 (ATCC CRL-2190), liver HepG2 (ECACC 85011430), and cardiac H9c2 (ECACC 88092904). Results revealed that MIC80 concentration (64 µg/mL) of Biochanin A exhibited a toxicity of 3.43 ± 1.24% against kidney cell lines, which is lower than the toxicity of the positive control, 108.27 µg/mL doxorubicin HCl, at 67.45 ± 8.16% (Figure 7A). Biochanin A also demonstrated low toxicity against cardiac cells, showing 4.64 ± 1.00% compared to doxorubicin HCl’s toxicity of 90.31 ± 4.90% at 92.7 µg/mL (Figure 7B). Meanwhile, Biochanin A induced low toxicity of 3.6 ± 0.68% against liver carcinoma cells compared to the toxicity of tamoxifen, which was 95.84 ± 2.59% at a concentration of 14.9 µg/mL (Figure 7C). Furthermore, Biochanin A did not exceed the threshold of 10% toxicity, indicating that it was safe for further drug development. The low toxicity profile of Biochanin A against normal cells was further supported by statistical analysis using a *t*-test with Welch’s correction, showing a significant difference compared to all the controls tested.

### 2.10. Structure-Activity Relationship (SAR) of Biochanin A Compared to Other Flavonoids

**Figure 8:**
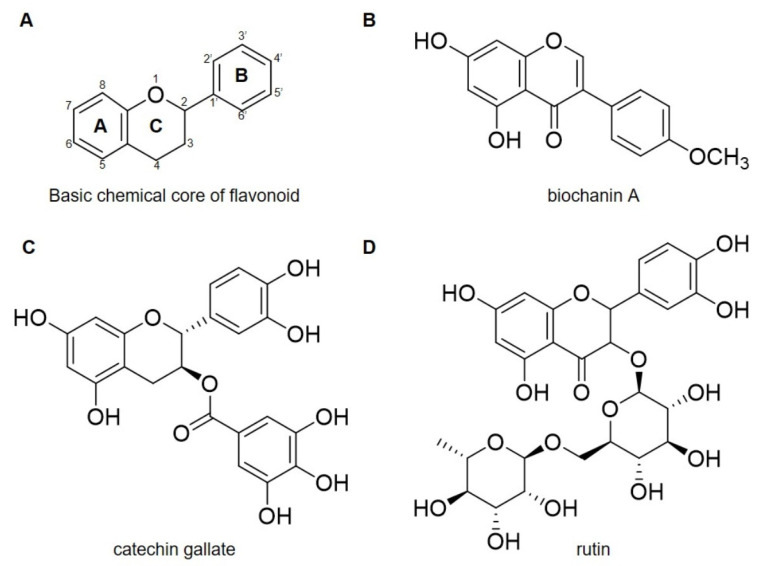
(**A**) the basic chemical core of flavonoid, (**B**) chemical structure of Biochanin A, (**C**) Catechin gallate, and (**D**) Rutin.

Biochanin A features a hydroxyl group at C-4’ on the B-ring and a methoxy group at C-5 on the A-ring (Figure 8A,B). These modifications directly affect Biochanin A’s interactions with FemX, FemA, and FemB. The methoxy group enhances the compound’s lipophilicity, aiding its interaction with the non-polar amino acid residues in the binding sites of both proteins [53,54]. This lipophilic modification improves membrane permeability, which is crucial for antibacterial activity, allowing Biochanin A to penetrate the bacterial cell wall effectively. The planar and compact structure of Biochanin A enables it to fit well within the binding pockets of FemX, FemA, and FemB, especially compared to bulkier compounds like catechin gallate and rutin, which can cause steric hindrance [55,56]. The hydroxyl group at C-4’ on the B-ring promotes hydrogen bonding, enhancing the binding affinity of Biochanin A for the active site residues of the target proteins. The combination of these modifications leads to highly efficient interactions with FemX, FemA, and FemB, enabling Biochanin A to exert its antibacterial effects [53,54].

Catechin gallate, on the other hand, is a flavan-3-ol that contains multiple hydroxyl groups on its aromatic rings (Figure 8C), which contribute to its strong hydrogen bonding capacity. These hydroxyl groups form hydrogen bonds with the active site residues of FemX, FemA, and FemB, enhancing catechin gallate’s high binding affinity for these proteins. The aromatic rings in catechin gallate also facilitate π-π stacking interactions with aromatic residues in the binding sites, further increasing its binding strength. Despite these advantages, catechin gallate’s flexible structure, due to the gallate ester at the C-3 position, can result in conformational strain [55]. This flexibility allows for some degree of interaction that may reduce binding specificity and potentially compromise the compound’s effectiveness in inhibiting FemX, FemA, and FemB. The gallate ester group adds additional flexibility, but it could also introduce structural instability, leading to strain-induced conformational changes that generate weak interactions with the target proteins [57].

Rutin, a flavonol, features a quercetin core and a sugar moiety (Figure 8D). The hydroxyl groups in the quercetin core enhance their strong binding affinity for FemX, FemA, and FemB by forming hydrogen bonds with key active site residues. However, the sugar moiety adds complexity to its interactions with the target proteins [56]. The polar nature of the sugar group allows for electrostatic interactions with polar or charged residues near the binding site, potentially increasing the compound’s binding affinity in specific contexts. Nevertheless, this polar group may also disrupt critical interactions necessary for effective inhibitory activity. The disaccharide moiety might cause steric hindrance, limiting rutin’s ability to engage with essential residues at the active sites of FemX, FemA, and FemB. Such steric hindrance can obstruct optimal alignment within the binding pockets, diminishing the overall binding affinity and potentially impacting the compound’s inhibitory potency [54,55,56].

Several key modifications to these flavonoid structures (Figure 8) influence their binding interactions with FemX, FemA, and FemB. The presence of hydroxyl groups generally enhances the hydrogen bonding capacity of these compounds, contributing to stronger interactions with active site residues. Specifically, hydroxyl groups on the B-ring improve the ability to form hydrogen bonds, while modifications such as the methoxy group in Biochanin A increase lipophilicity, facilitating membrane penetration [53]. The aromatic rings in catechin gallate and Biochanin A enable π-π stacking interactions with aromatic residues in the target proteins, further enhancing binding strength. However, modifications like the sugar moiety in rutin and the gallate ester in catechin gallate present challenges by causing steric hindrance or reducing binding specificity due to flexibility [54].

The balance among polarity, lipophilicity, hydrogen bonding, and steric effects primarily determines the overall antibacterial efficacy of these compounds. A compound with a highly lipophilic nature and a compact structure, such as Biochanin A, may demonstrate greater compatibility when interacting with hydrophobic residues and penetrating the bacterial membrane, potentially leading to effective inhibition of FemA and FemB. In contrast, compounds like catechin gallate, while capable of forming strong hydrogen bonds, may encounter limitations in binding specificity due to their structural flexibility. Rutin’s sugar moiety may enhance certain electrostatic interactions but might also hinder binding due to steric clashes with critical residues at the active site. Nonetheless, these in silico findings warrant further investigation particularly molecular dynamics to determine further the stability and interactions of Biochanin A in the active site of FemX, FemA, and FemB proteins [53,54].

## 3. Discussion

Plant-derived compounds, particularly flavonoids, play important protective roles in plants by mitigating oxidative stress, deterring insect predation, and limiting pathogen invasion [33,34,35]. These functions have led to sustained interest in flavonoids as sources of antibacterial agents. Numerous studies have reported antibacterial activity of flavonoid-containing plant extracts against both Gram-positive and Gram-negative bacteria [58,59,60,61]. For example, quercitrin and isoquercitrin from *Hypericum caprifoliatum* inhibit *Staphylococcus aureus* [62], while apigenin from *Portulaca oleracea* exhibits activity against *Salmonella typhimurium* [63]. Collectively, these studies indicate that flavonoids exert antibacterial effects through diverse mechanisms that are frequently strain-dependent, a feature commonly observed among polyphenolic natural products [64,65,66].

In line with these observations, the present study demonstrates that Biochanin A exhibits moderate but selective antibacterial activity against *S. aureus* ATCC 25923 and MDR strain *S. aureus* ATCC BAA-44, with MIC80 values of 64 µg/mL for both strains. These values are consistent with previously reported MIC ranges for Biochanin A against Gram-positive bacteria (32–128 µg/mL) [58,62], supporting the reproducibility of its antibacterial activity. While most previous studies on Biochanin A’s antibacterial activity focused on non-resistant strains or clinical isolates with variable resistance profiles [44,59], the present study is among the first to demonstrate that Biochanin A is active against *S. aureus* ATCC BAA-44, a multi-drug-resistant bacterium. This finding is particularly significant because strains like ATCC BAA-44 are notoriously difficult to treat due to their reported resistance to multiple conventional antibiotics [67].

In contrast, Biochanin A showed no measurable inhibitory activity against *S. aureus* ATCC 6538, with MIC80 values exceeding 512 µg/mL. Although ATCC 6538 is generally regarded as antibiotic-sensitive and is frequently used as a reference strain in antimicrobial testing, previous reports have noted that this strain can exhibit tolerance to certain non-antibiotic stressors and selected natural products [68,69]. Similar strain-specific insensitivity has been documented for other flavonoids, where compounds active against one *S. aureus* strain display limited or no activity against another [70,71,72]. These observations indicate that flavonoid susceptibility in *S. aureus* is influenced by strain-specific genetic factors aside from their antibiotic resistance profiles.

Our comparative in silico pan-genomic analysis has identified genes encoding an iron-hydroxamate ABC transporter substrate-binding protein, a major facilitator superfamily (MFS) transporter, and the peptide resistance ABC transporter activity modulator *VraH* as features unique to *S. aureus* ATCC 6538 (Figure 1). Transporter-associated genes have been implicated in adaptive responses to environmental stress and exposure to antibiotics in *S. aureus* and other Gram-positive bacteria [73,74,75]. Although these transporters are not directly linked to resistance against Biochanin A, their presence suggests potential mechanisms that may contribute to reduced susceptibility (Figure 1 and Table S1).

Iron-hydroxamate ABC transporters are involved in iron acquisition, which is essential for bacterial metabolism, stress tolerance, and virulence [76,77,78]. While there is no evidence that this transporter directly mediates flavonoid efflux, impaired iron uptake has been shown to increase bacterial susceptibility to oxidative and chemical stress, whereas efficient iron acquisition can enhance survival under adverse conditions [78]. Accordingly, the presence of this transporter may indirectly support bacterial fitness during Biochanin A exposure, consistent with previous studies linking iron homeostasis to antimicrobial tolerance [78,79].

The identification of an MFS transporter further supports a possible role for efflux-mediated tolerance. MFS transporters constitute one of the largest families of bacterial secondary transporters and have been associated with multidrug resistance through the export of structurally diverse compounds [40,41,42,43,44,45,46,47,48,49,50,51]. Several studies have demonstrated that MFS transporters contribute to bacterial resistance against plant-derived flavonoids, including luteolin, quercetin, galangin, naringenin, and apigenin [80]. Given the shared scaffold and similar physicochemical properties among these compounds, Biochanin A may be similarly recognized by MFS efflux systems [81,82]. However, direct experimental validation would be required to confirm this hypothesis.

The peptide resistance ABC transporter activity modulator *VraH* forms part of the *VraDEH* transporter system, which functions in conjunction with the *VraSR* two-component regulatory system to coordinate responses to cell envelope stress in *S. aureus* [83]. Activation of this system has been associated with reduced susceptibility to antibiotics and antimicrobial peptides targeting the bacterial membrane or cell wall, including daptomycin and gallidermin [84]. Given that Biochanin A induces membrane-associated stress, involvement of *VraDEH*-mediated adaptive responses may contribute to the reduced activity observed in ATCC 6538, although this remains speculative in the absence of functional validation.

Biochanin A has also been reported to exhibit selective activity within the gut microbiome, inhibiting pathogenic *Clostridia* while promoting the growth of beneficial Bifidobacteria [45]. Similar selective effects have been described for other flavonoids, supporting the view that strain selectivity is a common feature of flavonoid antibacterial activity rather than an isolated observation [45,47,48,66,70]. Such selectivity may be advantageous in minimizing disruption of beneficial microbial communities [71].

Consistent with previous reports, Biochanin A exhibited a bacteriostatic effect against MDR-SA. Comparable bacteriostatic profiles have been described for flavonoids such as epigallocatechin gallate, galangin, and 3-*O*-octanoyl-(+)-catechin [85,86,87], which induce cellular stress and membrane perturbation without rapid bactericidal activity [87,88]. Biochanin A’s activity profile resembles that of tetracycline based on bacterial viability assay (Results Section 2.3) [89]. However, variability in Biochanin A potency across MDR-SA strains has been reported previously, including weak or absent activity against certain clinical isolates [44], which is consistent with the strain-dependent effects observed in this study.

Recent studies have demonstrated that Biochanin A can enhance the activity of fluoroquinolones such as ofloxacin and ciprofloxacin against MDR-SA [44,90,91,92,93]. The present findings provide a plausible explanation for these observations, as compromised cell wall integrity may increase bacterial susceptibility to antibiotics. Similar adjuvant effects have been reported for other flavonoids that weaken cell wall or membrane stability [44,75]. These observations suggest that Biochanin A may be more appropriately considered as a potentiating agent rather than as a standalone antibacterial compound.

Additional insights into the adjuvant potential of Biochanin A can be gained by correlating its MIC80 against MDR-SA with its predicted in silico human steady-state volume of distribution (VDss) (Table S4). The VDss of Biochanin A is −0.341 in log L/kg, suggesting that the compound is largely confined to plasma and extracellular fluids, with limited distribution into body tissues (Table S4). A low VDss is considered favorable for treating bloodstream infections, as it indicates that a higher proportion of the drug remains in systemic circulation (Table S4) [94]. However, despite this favorable distribution profile, Biochanin A demonstrates poor systemic bioavailability [43], indicating that its plasma concentration following oral administration is significantly lower than the MIC80 required to inhibit MDR-SA. Consequently, the Biochanin A concentrations achieved through systemic administration are insufficient to exert effective antibacterial activity. Therefore, due to its relatively higher MIC80 (Results Section 2.1) and limited systemic exposure (Table S4), Biochanin A is unlikely to be effective as a standalone systemic antibiotic. Instead, it may hold greater promise in topical formulations or when used as an adjuvant to enhance the activity of existing antibiotics.

We also investigated the Biochanin A’s ability to disrupt the membrane integrity of MDR-SA using high-precision flow cytometry. Our findings demonstrate that Biochanin A effectively compromises the membrane permeability of most MDR-SA cells. A related study [95] showed that Biochanin A induces membrane damage in *Candida albicans*, leading to the uptake of propidium iodide. This uptake indicates compromised staphylococcal cell wall integrity due to interference with bacterial membrane synthesis as demonstrated in Results Section 2.5. Additionally, Biochanin A-treated cells displayed shrinkage and deformation in SEM analysis, suggesting a loss of structural integrity likely resulting from damage or alterations in cell wall structure [96]. These morphological changes support the hypothesis that Biochanin A’s antimicrobial activity primarily stems from its ability to disrupt the structural integrity of MDR-SA cells. These findings align with previous studies, which indicated that bacterial cell wall disruption is a common characteristic of compounds that primarily target cell wall-associated pathways [97].

Moreover, the observed phenotypic collapse in MDR-SA (Figure 4G–I) provides evidence that Biochanin A damages the bacterial cell wall due to the downregulation of critical genes involved in PG synthesis, such as *femA* and *femB* genes, as shown in Figure 5D. Multiple studies have highlighted the essential roles of these genes in PG biosynthesis [20,21,24,27,28,52,55,97,98,99] and their interaction with other antibiotic resistance-related genes supported by gene enrichment analyses illustrated in Figure S2. The enzyme FemX initiates the synthesis of the pentaglycine bridge in *S. aureus* by catalyzing the transfer of the first glycine residue from glycyl-tRNA to the ε-amino group of a lysine residue in the lipid II precursor [81,97,98,99,100]. FemA adds the second and third glycine residues to the pentaglycine bridge [82,99,100], while FemB contributes the fourth and fifth glycine residues to complete the bridge [81,82,100,101,102,103]. This pentaglycine bridge is critical for the function of penicillin-binding proteins (PBPs), including the β-lactam-insensitive PBP2a, which is necessary for cross-linking PG strands and maintaining the cell wall’s structural integrity [52].

**Figure 9:**
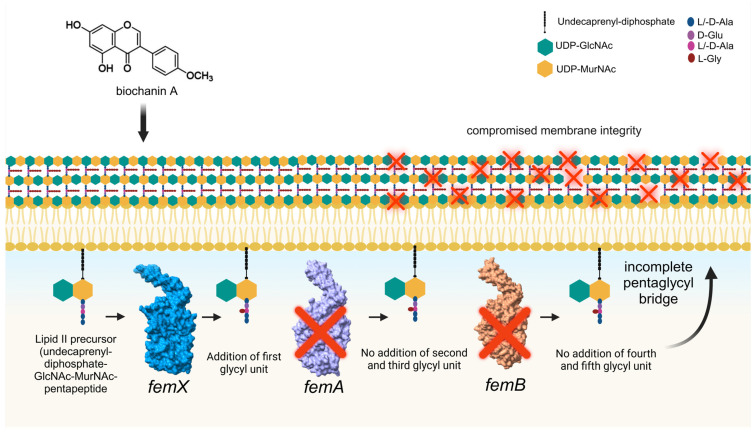
The proposed mechanism of action for Biochanin A involves its ability to disrupt a critical process in bacterial cell wall biosynthesis. The cross sign (X) indicates that the gene was downregulated significantly by Biochanin A. Created in BioRender. Dalisay, D. (https://BioRender.com/e39t921). [105].

Our proposed mechanism of action for Biochanin A, illustrated in Figure 9, suggests that Biochanin A does not target *femX* but significantly downregulates the genes *femA* and *femB*. This downregulation disrupts the biosynthesis of murein precursors, leading to the incomplete formation of the pentaglycine bridge [21,52]. Consequently, murein monomers retain only the initial glycine residue added by FemX, rendering them incapable of forming complete pentaglycine bridges [21,27,52,98,101,102,103]. These defective monomers cannot integrate into the PG matrix [99], thereby weakening the structure of the bacterial cell wall. This disruption creates a destabilized cell wall with impaired membrane permeability, as evidenced by PI uptake shown in Figure 4D. Although PBP2a may attempt to cross-link these defective monomers [100], the absence of functional pentaglycine bridges significantly undermines the structural integrity of the PG network [104]. Ultimately, this leads to a weakened cell wall and impaired membrane function, demonstrating the phenotypic outcomes in Figure 4G.

It is important to note that the transcriptional evidence supporting this proposed hypothesis remains indirect and requires further validation at the protein level. Specifically, protein-based analyses such as Western blotting to quantify FemA and FemB expression are necessary to confirm whether the observed transcriptional changes induced by Biochanin A translate into corresponding effects at the translational level.

Previous report indicated that the downregulation of *femA* and *femB* genes does not directly kill the pathogen [52,104], supporting our findings regarding the bacteriostatic effect of Biochanin A. However, the impaired PG matrix caused by the downregulation of *femA* and *femB* genes makes bacteria more susceptible to antibiotics [106]. This offers a strong explanation for why Biochanin A has demonstrated high effectiveness in studies investigating its synergistic effects [39,44,91,92]. Additionally, this is not the first time a compound from the flavonoid class has been investigated against FemX, FemA, and FemB proteins. Catechin gallate, a compound from the flavan-3-ol subclass, has been reported to have a high binding affinity for FemA protein [55], while rutin has shown in silico binding against FemX, FemA, and FemB proteins [56]. These two compounds and their derivatives have been reported to exhibit antibacterial activity against MDR-SA [86,87,88,107]. Although their binding affinities are greater than that of Biochanin A (Figure 6), no in vitro studies have yet been reported. Flavonoids such as Biochanin A, catechin gallate, and rutin share a common 15-carbon flavone skeleton (C6-C3-C6) (Figure 8), consisting of two benzene rings (A and B) linked by a three-carbon pyran ring (C) [86]. Despite the shared core structure, the structural variations among these compounds are crucial to their antibacterial activity, particularly against MDR-SA. Flavonoids are classified into distinct subgroups: for example, Biochanin A is an isoflavone, catechin gallate is a flavan-3-ol, and rutin is a flavonol (Figure 8). Their differing binding affinities against FemX, FemA, and FemB proteins (Figure S3) suggest that additions and modifications to the chemical structure of the flavonoid scaffold strongly influence binding affinities, which may translate into antibacterial activity [86,87].

Furthermore, the safety of Biochanin A as an antibacterial drug candidate was established using a combination of in vitro and in silico approaches (Figure 7 and Table S4). Although Biochanin A exhibited lower binding affinities to FemA and FemB proteins compared to catechin gallate and rutin, its potential remains as a promising drug candidate due to its favorable in silico ADMET properties (Table S4). This apparent contradiction underscores the complexity of drug development, where strong target binding affinity does not necessarily correlate with clinical efficacy. In molecular docking analyses (Figure 6 and Table S3), Biochanin A showed weaker binding affinities against FemA and FemB proteins, indicating limited inhibitory potential at the molecular level (Table S3). However, pharmacokinetics and other drug-like properties are equally critical determinants of therapeutic success.

Notably, in silico ADMET predictions suggest that Biochanin A complies with Lipinski’s Rule of Five, indicating good oral bioavailability. It also demonstrates high Caco-2 permeability and favorable human intestinal absorption, supporting its potential for efficient oral uptake (Table S4). In contrast, catechin gallate and rutin, despite exhibiting stronger binding affinities, suffer from poor gastrointestinal absorption, which may compromise their systemic bioavailability. Moreover, the lower VDss for Biochanin A suggests a preference for remaining in plasma, thereby concentrating therapeutic activity in circulation and minimizing off-target exposure (Table S4). Biochanin A’s low unbound drug fraction also indicates the potential for sustained plasma levels, while its classification as a non-inhibitor of P-glycoproteins suggests a lower likelihood of drug-drug interactions that could impair absorption. Additionally, Biochanin A’s high predicted clearance rate indicates efficient elimination, which may help prevent drug accumulation and reduce the risk of long-term toxicity (Table S4).

Despite these promising in silico predictions, experimental validation is essential. In vitro studies, including Caco-2 permeability assays, are needed to confirm intestinal absorption. Also, liver microsome stability studies are necessary to evaluate metabolic stability. In addition, we conducted in vitro cytotoxicity assays in normal mammalian cell lines (kidney, liver, heart), which demonstrated that Biochanin A is non-toxic at concentrations corresponding to its MIC80 values, confirming a favorable therapeutic potential. However, in vivo pharmacokinetic analyses are required to further assess systemic exposure, distribution, and safety, supporting the translation of these findings into potential clinical applications.

Importantly, previous reports have demonstrated organ-protective effects of Biochanin A, including hepatoprotection through the preservation of cellular integrity and membrane stability [108], attenuation of TAC-induced cardiac fibrosis, inflammation, oxidative stress, and hypertrophy [109], as well as nephroprotection via downregulation of apoptotic markers caspase 3 and p53 [110]. Collectively, these data provide additional reassurance of Biochanin A’s safety and potential applicability as an antibacterial agent, particularly as an adjuvant or topical treatment.

## 4. Materials and Methods

### 4.1. Standards and Biological Samples

The standard Biochanin A (98% pure) was obtained from Changzhou Guiding Bio-Tech. Co., Ltd. (Changzhou, China). Positive controls used in this study were tetracycline (Sigma-Aldrich Co.^®^, St. Louis, MO, USA). Dimethyl sulfoxide (DMSO) (Bangkok, Thailand) was used as the negative control. Biological standards such as multidrug-resistant strain *S. aureus* ATCC BAA-44 and non-resistant strains *S. aureus* ATCC 6538 and ATCC 25923 were purchased from the American Type Culture Collection (ATCC) (Manassas, VA, USA). The cell lines used for toxicity testing were human renal proximal tubule epithelial cell line (HK-2; ATCC CRL-2190), human hepatocellular carcinoma (HepG2; ECACC 85011430), and rat cardiomyocytes (H9c2 [2-1]; ECACC 88092904) were secured from Sigma-Aldrich^®^ (Sigma-Aldrich Co., St. Louis, MO, USA). The total RNA Extraction Kit was purchased from Vivantis Technologies (Kuala Lumpur, Malaysia), and the cDNA synthesis kit was obtained from Takara PrimeScript™ (San Jose, CA, USA). SYBR^®^ Master Mix was secured from KAPA Biosystems (Wilmington, MA, USA).

### 4.2. Minimum Inhibitory Concentration (MIC) Testing

The minimum inhibitory concentration (MIC) assay was conducted following the Clinical and Laboratory Standards Institute (CLSI) guidelines [111]. An overnight bacterial culture was transferred to fresh Mueller-Hinton Broth (MHB), and the bacterial concentration was adjusted to 1 × 10^6^ CFU/mL. Biochanin A was prepared and serially diluted two-fold in a 96-well plate to obtain final concentrations of 512, 256, 128, 64, 32, 16, 8, 4, and 2 µg/mL. Tetracycline was used as a positive control due to its well-documented antibacterial activity against various *S. aureus* strains and its established role as a reference standard in antibacterial testing. Meanwhile, dimethyl sulfoxide (DMSO) served as the negative control, as it was the solvent used to dissolve Biochanin A. This control was included to ensure that any observed antibacterial activity was attributable to Biochanin A itself and not to DMSO, thereby excluding the possibility that the solvent alone produced inhibitory effects. The treated bacterial suspensions were incubated at 37 °C for 18 to 24 h. Absorbance was measured at 600 nm using a microplate reader spectrophotometer (BMG Labtech Clariostar^®^, Ortenberg, Germany), and the percentage bacterial growth inhibition was calculated using Equation (1). The experiment was performed in triplicate across three independent trials. The bacterial inhibition > 80% was considered as the MIC value and annotated as MIC80.

(1) $$\%\;\mathrm{growth}\;\mathrm{of}\;\mathrm{inhibition}=\left(\frac{\left(\mathrm{Negative}\;\mathrm{control}\;\mathrm{Absorbance}\;-\;\mathrm{Sample}\;\mathrm{Absorbance}\right)}{\mathrm{Negative}\;\mathrm{control}\;\mathrm{Absorbance}}\right)\times 100,$$

### 4.3. Comparative Genome Analysis

To provide a deeper understanding of the genomic differences among three ATCC standard *S. aureus* strains, namely ATCC BAA-44, ATCC 25923, and ATCC 6538, we focused on the comparative analysis of genes associated with transporter systems using the Efficient Database framework for Comparative Genome Analyses based on BLAST Score Ratios (EDGAR v3.5) (https://edgar3.computational.bio.uni-giessen.de/cgi-bin/edgar.cgi, accessed on 30 December 2025) [112]. Transporter genes are recognized biomarkers of antibiotic resistance in *S. aureus* [51]; hence, their pan-genome data were retrieved in .xcl format from the “EDGAR_*Staphylococcus*” genomic subset (Table S1). Subsequently, *S. aureus* ATCC 6538 was selected as the reference genome for iterative comparisons, as it exhibited resistance to Biochanin A in antibacterial testing. To illustrate the distribution and shared transporter genes among the strains, a chord diagram was generated using Circos Table Viewer (https://mk.bcgsc.ca/tableviewer/visualize/, accessed on 30 December 2025) to provide a visual representation of overlap and strain-specific transporter genes [113].

### 4.4. Bacterial Cell Viability Assay Using Spread Plate Method

The spread plate method was employed to assess the efficacy of Biochanin A in reducing the viability of *S. aureus* ATCC BAA-44 following treatment exposure. A bacterial suspension was prepared in phosphate-buffered saline (PBS) and the optical density was adjusted to 0.5 at 600 nm before exposure to Biochanin A at 64 µg/mL. DMSO served as a negative control. The treated bacterial cells were incubated for 6 h in a shaking incubator at 32 °C. Then, 10 µL from each treatment was aliquoted and serially diluted in 100 µL of PBS, subsequently diluted to achieve a dilution factor of 10^6^. Twenty microliters of diluted bacterial cells were then plated on tryptone soy agar (TSA) and incubated for 24 h. After incubation, colony-forming units per mL (CFU/mL) were quantified using Equation (2) and statistically analyzed via one-way ANOVA followed by Tukey’s test in GraphPad Prism v.9.3.1 (San Diego, CA, USA). The experiment was conducted in triplicate in three independent trials.

(2) $$\frac{\mathrm{CFU}}{\mathrm{mL}}=\frac{\mathrm{no}.\;\mathrm{of}\;\mathrm{colonies}\;\times \;\mathrm{dilution}\;\mathrm{factor}}{\mathrm{volume}\;\mathrm{of}\;\mathrm{culture}\;\mathrm{plated}\;(\mathrm{mL})},$$

### 4.5. Live/Dead Propidium Iodide and Calcein Uptake Assay Using Flow Cytometer

The Amnis^®^ FlowSight^®^ Imaging Flow Cytometer (Fremont, CA, USA) was used to assess the live/dead ratio of *S. aureus* ATCC BAA-44 treated with Biochanin A at 64 µg/mL. The Amnis^®^ FlowSight^®^ flow cytometry system combines high-precision imaging with fluorescence detection to provide a detailed analysis of bacterial populations [114]. In this experiment, a live/dead cell double staining kit containing propidium iodide (PI) and calcein acetoxymethyl (CA) (Sigma-Aldrich Co., St. Louis, MO, USA) wherein 5 μL of CA and 20 μL of PI were mixed with 24 mL of PBS. The system analyzed 10,000 bacterial events, gating the bacterial populations into four regions based on their fluorescence signals: Region 1 (R1) with a strong PI signal indicating dead cells with disrupted membranes; Region 2 (R2) with double-stained cells, signifying compromised membrane integrity and partial viability; Region 3 (R3) with a strong CA signal representing live, metabolically active cells; and Region 4 (R4) containing cell debris and other nonviable particles with undefined fluorescence. In this experiment, 70% Ethanol (*v*/*v*) served as a positive control, while DMSO was used as a negative control. The experiment was conducted in triplicate across three independent trials. The cellular events of each region were statistically analyzed per treatment using one-way ANOVA followed by Tukey’s test in GraphPad Prism v.9.3.1 (San Diego, CA, USA).

### 4.6. Fluorescence Microscopy

To complement the quantitative flow cytometry data, fluorescence microscopy was conducted to qualitatively assess the physiological state of *S. aureus* ATCC BAA-44 after treatment with Biochanin A at 64 µg/mL. PI and CA dye solutions were prepared similarly to the live/dead PI and CA uptake assay to visualize live and dead cells. Live cells fluoresce green due to the conversion of CA into fluorescent calcein by intracellular esterase activity, indicating metabolic activity and intact membrane integrity. Dead cells fluoresce red as PI penetrates damaged membranes and intercalates with intracellular DNA [114]. The stained bacterial suspension was prepared in PBS and mounted on a glass slide prior to observation under a fluorescence microscope (IX38 Olympus) (Evident Scientific Inc, Waltham, MA, USA) at 100× magnification. To enhance the visualizations of fluorescent signals, images were captured at 100× magnification and then digitally expanded (200% zoom) during analysis (Figure S1). The scale bar represents the 100× magnification.

### 4.7. Scanning Electron Microscopy (SEM)

SEM analysis was conducted to investigate the effect of Biochanin A on the morphology of *S. aureus* ATCC BAA-44 after treatment. MDR-SA cells were exposed to Biochanin A at a concentration of 64 µg/mL for 24 h, and then fixed with 2.5% glutaraldehyde for 4 h at 4 °C in a chiller. The fixed bacterial cells were gradually dehydrated using 30%, 50%, 85%, 95%, and absolute ethanol. The dehydrated cells were mounted on carbon tape and placed in an aluminum stub (JEOL Ø25 × 10 mm cylinder SEM sample stub) (Tokyo, Japan), followed by 30 s of sputter coating with gold using the JEOL Smart Coater^TM^ (Tokyo, Japan) to enhance sample conductivity. Morphology was examined at a magnification of 30,000× *g* using the JCM-7000 NeoScope™ Benchtop SEM (Tokyo, Japan) in high vacuum mode. The diameter of the treated bacteria was measured to detect any variations in morphological size across treatments. Finally, the bacterial size for each treatment was statistically analyzed using one-way ANOVA followed by Tukey’s test in GraphPad Prism v.9.3.1 (San Diego, CA, USA).

### 4.8. Relative Gene Expression Analysis

To evaluate the effect of Biochanin A on the gene expression of *femX*, *femA*, and *femB*, a quantitative reverse transcription polymerase chain reaction (qRT-PCR) assay was performed. A 5 mL suspension of *S. aureus* ATCC BAA-44 was grown in MHB to an optical density of 0.5 at 600 nm. The bacterial cells were exposed to Biochanin A at a concentration of 64 µg/mL and incubated at 37 °C for 6 h [48]. Following the incubation period, the bacterial cells were centrifuged at 3700 centrifugal force (× *g*) for 10 min and washed twice with PBS to remove residual media. Total RNA was extracted using the Vivantis GF-1 Total RNA Extraction Kit (Kuala Lumpur, Malaysia), following the manufacturer’s protocol. To synthesize complementary DNA (cDNA) from the extracted RNA, reverse transcription was performed using the Takara PrimeScript™ 1st Strand cDNA Synthesis Kit (San Jose, CA, USA) following the manufacturer’s protocol. The qPCR was conducted using the Bioer Line Gene 9600 Real-Time PCR Detection System (Hangzhou, China) to amplify *femX*, *femA*, and *femB* using gene primers [104] (Table S2). The amplification reactions were prepared using the KAPA SYBR^®^ FAST qPCR Master Mix (2X) Universal (Wilmington, MA, USA). The expression levels of *femX*, *femA*, and *femB* were normalized against the reference gene 16S rRNA [115], a widely accepted housekeeping gene in bacterial studies. Relative expression levels were calculated using the Livak method stated in Equation (3) [116]. The experiment was conducted in triplicate across three independent trials. The sample means of expressed genes were statistically analyzed using an unpaired *t*-test with Welch’s correction in GraphPad Prism v.9.3.1 (San Diego, CA, USA), where a significant difference in the sample means is indicated by a *p*-value of < 0.05.

(3) $$\mathrm{Gene}\;\mathrm{expression}=2^{-∆∆\mathrm{Ct}},$$

### 4.9. In Silico Analyses

In silico protein-ligand binding analysis was performed using AutoDock Tools v1.5.7 [117]. The protein structures (.pdb format) of FemX (6SNR) and FemA (1LRZ) were obtained from the PDB, while the FemB (Q2FYR1) structure was sourced from the UniProt database. The obtained proteins were prepared by removing water molecules and adding polar hydrogens. Meanwhile, the ligand Biochanin A (.sdf) was acquired from PubChem [118] and its geometry was optimized using Avogadro v1.1.2.0, setting the torsional degrees of freedom (TORSDOF) to 4 with default rotational bonds. The Merck Molecular Force Field 94 (MMFF94) was applied using a steepest descent algorithm for 500 steps and a convergence of 10^−7^. In this experiment, catechin gallate and rutin were used as controls, as these flavonoid compounds exhibit antibacterial activity against MDR-SA [57,119]. Their mechanisms have recently been explored for binding interactions against FemX, FemA [55], and FemB [56]. The interacting residues of the active site of FemX were located at LYS33, TRP38, ARG208, TYR212, and TYR320 [98]. Conversely, the interacting residues of the FemA active site were identified as GLY330, LYS383, PHE363, and ASP150, while those of FemB included LYS106, GLN155, THR153, TYR70, SER153, and ASP33 [56]. The grid coordinates of the active sites were as follows: for FemX, x-center: 44.082, y-center: −1. 82, z-center: 51.537; for FemA, x-center: 42.228, y-center: 53.091, z-center: 95.003; and for FemB, x-center: 16.52, y-center: −5.086, z-center: −6.788. Both grids measured 50 Å × 50 Å × 50 Å. The spacing value was set to 0.500 Å, while the binding affinity with the lowest root-mean square deviation (RMSD ≤2.0 Å) was selected for post-analysis using Biovia Discovery Studio Visualizer v24.1.0.23298 and UCSF ChimeraX v1.6.1. The molecular docking experiment involved 32 exhaustiveness runs, repeated 3 times to obtain the ± SEM. Statistical analyses of the binding affinities of Biochanin A against FemX, FemA, and FemB proteins were conducted using a *t*-test with Welch’s correction, while the comparison of binding affinities among Biochanin A, catechin gallate, and rutin was performed using one-way ANOVA with Tukey’s test. A statistical difference was considered significant when the *p*-value was < 0.05. Furthermore, the pharmacokinetics, ADMET, and druggability of Biochanin A were analyzed in silico using pkCSM-pharmacokinetics (https://biosig.lab.uq.edu.au/pkcsm/, accessed on 30 December 2025) [120].

### 4.10. Toxicity Screening by Lactate Dehydrogsenase Assay

The lactate dehydrogenase toxicity assay was conducted to assess the damage induced by the compound on normal cell lines, as measured by the release of the lactate dehydrogenase enzyme [114]. Biochanin A was tested at 64 µg/mL for toxicity against HK-2 (kidney), HepG2 (liver), and H9c2 [2-1] (cardiac) cell lines. Doxorubicin hydrochloride (Sigma-Aldrich Co., St. Louis, MO, USA) served as the positive control for the HK-2 and H9c2 cell lines at 108.7 and 92.7 µg/mL, respectively. Meanwhile, tamoxifen (Sigma-Aldrich, USA) at 14.9 µg/mL was utilized as the positive control for the HepG2 cell line. The negative control for the experiment was 0.1% DMSO. The cell seeding concentration for all three cell lines was set at 5000 cells/well, followed by a pre-treatment incubation period of 2–4 h to facilitate cellular attachment. Treatment was applied prior to an 18 h incubation before performing the LDH toxicity assay. LDH absorbance was measured at 490 nm using the multimode microplate reader (Clariostar^®^, BMG Labtech GmbH, Ortenberg, Germany). A threshold of 10% was established in the experiment to determine the lowest minimum compound efficacy against the tested cell lines. Any values exceeding the 10% baseline were deemed toxic. The experiment was conducted in triplicate across three independent trials. The percent toxicity was calculated using Equation (4). The results were analyzed and illustrated using GraphPad Prism v9.3.1 for Windows (GraphPad Software, San Diego, CA, USA).

(4) $$\%\mathrm{Toxicity}=\frac{\left(\mathrm{Experimental}\;\mathrm{Control}-\mathrm{Experimental}\;\mathrm{Blank}\right)\;-\;\left(\mathrm{Negative}\;\mathrm{Control}-\mathrm{Negative}\;\mathrm{Control}\;\mathrm{blank}\right)}{\left(\mathrm{High}\;\mathrm{Control}\;\mathrm{Value}-\mathrm{High}\;\mathrm{Control}\;\mathrm{blank}\right)\;-\;\left(\mathrm{Low}\;\mathrm{Control}\;\mathrm{Value}-\mathrm{Low}\;\mathrm{Control}\;\mathrm{blank}\right)}\;\times \;100,$$

## 5. Conclusions

The findings of this study demonstrate that Biochanin A exhibits antibacterial activity against MDR-SA, specifically *S. aureus* ATCC BAA-44. Flow cytometry analyses indicate that Biochanin A compromises bacterial membrane integrity, while SEM observations reveal structural alterations in MDR-SA morphology. Additionally, Biochanin A downregulates *femA* and *femB*, genes involved in synthesizing PG pentaglycine residues, suggesting a potential disruption of bacterial cell wall synthesis. In silico and in vitro toxicological assessments indicate no significant toxicity to the liver, heart, or kidneys under the tested conditions.

These results provide preliminary mechanistic insights into the molecular antibacterial activity of Biochanin A. Further studies are warranted to explore its efficacy in vivo, potential synergistic effects with other antibacterial agents, and the impact on additional bacterial biosynthesis pathways. While these findings highlight the promise of Biochanin A as a plant-derived antibacterial candidate, the translational potential requires further experimental validation.

## Data Availability

The original contributions presented in this study are included in the article. Further inquiries can be directed to the corresponding authors.

## Supplementary Materials

The following supporting information can be downloaded at: https://www.mdpi.com/article/10.3390/antibiotics15020195/s1. Table S1. Comparative genome analysis of transporter genes presents in *S. aureus* ATCC 6538, aureus ATCC25923, and *S. aureus* ATCC BAA-44 revealed that only *S. aureus* ATCC 6538. Table S2. Primer sequence of target genes *femX*, *femA* and *femB* and reference gene 16S rRNA. Table S3. Binding interaction of Biochanin A, catechin gallate, and rutin against *femX*, *femA*, and *femB*. Table S4. Pharmacokinetics of Biochanin A compared to other flavonoids targeting *femA* and *femB*. Figure S1. Fluorescent microscopy at 200× zoom of MDR-SA treated with 70% Ethanol (*v*/*v*), DMSO, and Biochanin A. Figure S2. Gene enrichment analyses of *femA* and *femB* showed its association to other genes that play a key role in other biosynthesis pathways.

## Abbreviations

The following abbreviations are used in this manuscript:

AMR	antimicrobial resistance
BBB	blood–brain barrier
CFU/mL	colony-forming units per milliliter
CNS	central nervous system
DMSO	Dimethyl sulfoxide
*femA*	factor essential for methicillin resistance A gene
*femB*	factor essential for methicillin resistance B gene
*femX*	factor essential for methicillin resistance X gene
Fu	Fraction unbound
LDH	lactate dehydrogenase
MDR-SA	multidrug-resistant *S. aureus*
MIC	minimum inhibitory concentration
NS	no significance
OD	optical density
PBP2a	Penicillin-Binding Protein 2a
PBS	phosphate-buffered saline
PG	peptidoglycan
CA	Calcein AM
PI	propidium iodide
SAR	structure–activity relationship
SEM	scanning electron microscopy
±SEM	standard error of the mean
TSA	tryptone soy agar
WHO	World Health Organization
