# Supplementary material for: Biochanin A, a Plant Isoflavone, Disrupts Peptidoglycan Biosynthesis by Downregulating femA and femB, and Impairs Cell Wall Integrity in Multidrug-Resistant Staphylococcus aureus
Source: Antibiotics (Basel). 2026 Feb 10;15(2):195. doi: 10.3390/antibiotics15020195 (PMC12937266; doi:10.3390/antibiotics15020195)
Supplement: Supplementary file 1 [file antibiotics-15-00195-s001.zip › antibiotics-4020209-supplementary.pdf]

Biochanin A, a Plant Isoflavone, Disrupts Peptidoglycan Biosynthesis by Downregulating *femA* and *femB*, and Impairs Cell Wall Integrity in Multidrug-Resistant *Staphylococcus aureus*

Jade Joshua R. Teodosio[1], Kathryn Ann H. Dizon[1], Julyanna R. Bruna[1], Jan Vincent N. Sollesta[2], Zenith M. Villorente[2], Jonel P. Saludes[3,4,5], and Doralyn S. Dalisay[1,5,6*]

[1] Center for Chemical Biology and Biotechnology (C2B2), University of San Agustin, Iloilo City 5000, Philippines; jjteodosio@usa.edu.ph; kadizon@usa.edu.ph; julyanna@usa.edu.ph

[2] Maridan Industries, Inc., Jaro, Iloilo City 5000, Philippines; jvsollesta@maridan.com.ph; zmvillorente@maridan.com.ph

[3] Center for Natural Drug Discovery and Development (CND3), University of San Agustin, Iloilo City 5000, Philippines; jsaludes@usa.edu.ph

[4] Department of Chemistry, University of San Agustin, Iloilo City, Philippines; jsaludes@usa.edu.ph

[5] Balik Scientist Program, Department of Science and Technology-Philippine Council for Health Research and Development, Taguig City, Philippines; jsaludes@usa.edu.ph; ddalisay@usa.edu.ph

[6] Department of Biology, University of San Agustin, Iloilo City, Philippines; ddalisay@usa.edu.ph

[*] Correspondence: ddalisay@usa.edu.ph; Tel.: +63-33-501-0350

SUPPLEMENTARY INFORMATION

List of Supporting Tables **Page**

**Table S1**	Comparative genome analysis of transporter genes presents in *S. aureus* ATCC 6538, aureus ATCC25923, and *S. aureus* ATCC BAA-44 revealed that only *S. aureus* ATCC 6538.	**2-13**
**Table S2**	Primer sequence of target genes *femX*, *femA* and *femB* and reference gene 16S rRNA.	**14**
**Table S3**	Binding interaction of BCA, catechin gallate, and rutin against *femX*, *femA*, and *femB*.	**15-16**
**Table S4**	Pharmacokinetics of BCA compared to other flavonoids targeting *femA* and *femB*.	**17**

List of Supporting Figures **Page**

**Figure S1**	Fluorescent microscopy at 200X zoom of MDRSA treated with 70% Ethanol, DMSO, and BCA.	**18**
**Figure S2**	Gene enrichment analyses of femA and femB showed its association to other genes that play a key role in other biosynthesis pathway.	**19**

Table S1. Comparative analysis of 236 transporter genes in *S. aureus* ATCC 6538, ATCC BAA-44, and ATCC 25923.

Transporter Gene No.	Gene Name	*S. aureus* ATCC 6538	*S. aureus* ATCC BAA-44	*S. aureus* ATCC 25923
1	B4602_RS00055, ,psos,AzlC family ABC transporter permease	Present	Present	Present
2	B4602_RS00795, ,psos,efflux RND transporter periplasmic adaptor subunit	Present	Present	Absent
3	B4602_RS00315, ,psos,Na/Pi cotransporter family protein	Present	Present	Present
4	B4602_RS00325, ,psos,DMT family transporter	Present	Present	Absent
5	B4602_RS00360, ,psos,staphyloferrin B ABC transporter permease subunit SirC	Present	Present	Present
6	B4602_RS00365, ,psos,staphyloferrin B ABC transporter permease subunit SirB	Present	Present	Present
7	B4602_RS00370, ,psos,staphyloferrin B ABC transporter substrate-binding protein SirA	Present	Present	Present
8	B4602_RS00390, ,psos,staphyloferrin B export MFS transporter	Present	Present	Present
9	B4602_RS00430, ,psos,MFS transporter	Present	Present	Present
10	B4602_RS00490, ,psos,tetracycline efflux MFS transporter Tet(38)	Present	Present	Present
11	B4602_RS00505, ,psos,phosphonate ABC transporter, permease protein PhnE	Present	Present	Present
12	B4602_RS00510, ,psos,phosphonate ABC transporter, permease protein PhnE	Present	Present	Present
13	B4602_RS00515, ,psos,phosphonate ABC transporter ATP-binding protein	Present	Present	Present
14	B4602_RS00520, ,psos,phosphate/phosphite/phosphonate ABC transporter substrate-binding protein	Present	Present	Present
15	B4602_RS00645, ,psos,cation transporter	Present	Present	Present
16	B4602_RS00665, ,psos,ABC transporter ATP-binding protein	Present	Present	Present
17	B4602_RS00670, ,psos,ABC transporter substrate-binding protein	Present	Present	Present
18	B4602_RS00675, ,psos,ABC transporter permease	Present	Present	Present

Table S1. Comparative analysis of 236 transporter genes in *S. aureus* ATCC 6538, ATCC BAA-44, and ATCC 25923.

Transporter Gene No.	Gene Name	*S. aureus* ATCC 6538	*S. aureus* ATCC BAA-44	*S. aureus* ATCC 25923
19	B4602_RS00695, ,psos,MFS transporter	Present	Present	Present
20	B4602_RS00305, ,psos,MFS transporter	Present	Present	Present
21	B4602_RS00800, ,psos,ABC transporter ATP-binding protein	Present	Present	Absent
22	B4602_RS00825, ,psos,ABC transporter ATP-binding protein	Present	Present	Absent
23	B4602_RS00835, ,psos,ABC transporter ATP-binding protein	Present	Present	Present
24	B4602_RS00840, ,psos,ABC transporter permease	Present	Absent	Present
25	B4602_RS00845, ,psos,ABC transporter permease	Present	Present	Present
26	B4602_RS00850, ,psos,peptide ABC transporter substrate-binding protein	Present	Present	Present
27	B4602_RS00875, ,psos,sn-glycerol-3-phosphate ABC transporter ATP-binding protein UgpC	Present	Present	Present
28	B4602_RS00885, ,psos,sugar ABC transporter permease	Present	Present	Present
29	B4602_RS00890, ,psos,sugar ABC transporter permease	Present	Present	Present
30	B4602_RS01000, ,psos,ABC transporter substrate-binding protein	Present	Present	Present
31	B4602_RS01035, ,psos,PTS transporter subunit EIIC	Present	Present	Present
32	B4602_RS01055, ,psos,PTS sugar transporter subunit IIA	Present	Present	Present
33	B4602_RS01060, ,psos,PTS sugar transporter subunit IIB	Present	Present	Present
34	B4602_RS01065, ,psos,PTS galactitol transporter subunit IIC	Present	Present	Present
35	B4602_RS01195, ,psos,ribose transporter RbsU	Present	Present	Present
36	B4602_RS01210, ,psos,MFS transporter	Present	Present	Present
37	B4602_RS01225, ,psos,ABC transporter ATP-binding protein	Present	Present	Present
38	B4602_RS01230, ,psos,ABC transporter permease	Present	Present	Present
39	B4602_RS01235, ,psos,ABC transporter permease	Present	Present	Present
40	B4602_RS01390, ,psos,formate/nitrite transporter family protein	Present	Present	Absent
41	B4602_RS01415, ,psos,ABC transporter permease	Present	Present	Present
42	B4602_RS01420, ,psos,ABC transporter ATP-binding protein	Present	Present	Present

Table S1. Comparative analysis of 236 transporter genes in *S. aureus* ATCC 6538, ATCC BAA-44, and ATCC 25923.

Transporter Gene No.	Gene Name	*S. aureus* ATCC 6538	*S. aureus* ATCC BAA-44	*S. aureus* ATCC 25923
43	B4602_RS01445, ,psos,NupC/NupG family nucleoside CNT transporter	Present	Present	Present
44	B4602_RS01530, ,psos,PTS ascorbate transporter subunit IIC	Present	Present	Present
45	B4602_RS01535, ,psos,PTS sugar transporter subunit IIB	Present	Present	Present
46	B4602_RS01540, ,psos,PTS sugar transporter subunit IIA	Present	Present	Present
47	B4602_RS01555, ,psos,multidrug efflux transporter transcriptional repressor MepR	Present	Present	Present
48	B4602_RS01560, ,psos,multidrug efflux MATE transporter MepA	Present	Present	Present
49	B4602_RS01570, ,psos,glycerol-3-phosphate transporter	Present	Present	Present
50	B4602_RS01645, ,psos,ABC transporter ATP-binding protein	Present	Present	Present
51	B4602_RS01650, ,psos,ABC-2 transporter permease	Present	Present	Present
52	B4602_RS01820, ,psos,L-cystine transporter	Present	Present	Present
53	B4602_RS02130, ,psos,sodium-dependent transporter	Present	Present	Present
54	B4602_RS02145, ,psos,methionine ABC transporter ATP-binding protein	Present	Present	Present
55	B4602_RS02150, ,psos,ABC transporter permease	Present	Present	Present
56	B4602_RS02155, ,psos,dipeptide ABC transporter glycylmethionine-binding lipoprotein	Present	Present	Present
57	B4602_RS02575, ,psos,NupC/NupG family nucleoside CNT transporter	Present	Present	Present
58	B4602_RS02850, ,psos,MFS transporter	Present	Present	Present
59	B4602_RS03100, ,psos,ABC transporter substrate-binding protein	Present	Present	Present
60	B4602_RS03105, ,psos,iron ABC transporter permease	Present	Present	Present
61	B4602_RS03140, ,psos,DMT family transporter	Present	Present	Present
62	B4602_RS03200, ,psos,metal ABC transporter substrate-binding protein	Present	Present	Present
63	B4602_RS03205, ,psos,metal ABC transporter permease	Present	Present	Present

Table S1. Comparative analysis of 236 transporter genes in *S. aureus* ATCC 6538, ATCC BAA-44, and ATCC 25923.

Transporter Gene No.	Gene Name	*S. aureus* ATCC 6538	*S. aureus* ATCC BAA-44	*S. aureus* ATCC 25923
64	B4602_RS03210, ,psos,metal ABC transporter ATP-binding protein	Present	Present	Present
65	B4602_RS03230, ,psos,teichoic acids export ABC transporter ATP-binding subunit TagH	Present	Present	Present
66	B4602_RS03235, ,psos,teichoic acids export ABC transporter permease subunit TagG	Present	Present	Present
67	B4602_RS03260, ,psos,ABC transporter ATP-binding protein	Present	Present	Present
68	B4602_RS03280, ,psos,ABC transporter ATP-binding protein	Present	Present	Present
69	B4602_RS03285, ,psos,iron ABC transporter permease	Present	Present	Present
70	B4602_RS03290, ,psos,iron ABC transporter permease	Present	Present	Present
71	B4602_RS03355, ,psos,ABC transporter ATP-binding protein	Present	Present	Present
72	B4602_RS03370, ,psos,inorganic phosphate transporter	Present	Present	Present
73	B4602_RS03425, ,psos,MFS transporter	Present	Present	Present
74	B4602_RS03480, ,psos,ABC transporter ATP-binding protein/permease	Present	Present	Present
75	B4602_RS03485, ,psos,amino acid ABC transporter ATP-binding/permease protein	Present	Present	Present
76	B4602_RS03535, ,psos,multidrug efflux MFS transporter NorA	Present	Present	Present
77	B4602_RS03560, ,psos,PTS transporter subunit EIIA	Present	Present	Present
78	B4602_RS03570, ,psos,HlyC/CorC family transporter	Present	Present	Present
79	B4602_RS03670, ,psos,ABC transporter ATP-binding protein	Present	Present	Present
80	B4602_RS03675, ,psos,ABC transporter permease/substrate-binding protein	Present	Present	Present
81	B4602_RS03710, ,psos,peptide MFS transporter	Present	Present	Present
82	B4602_RS03725, ,psos,DMT family transporter	Present	Present	Present
83	B4602_RS03765, ,psos,ABC transporter permease	Present	Present	Present
84	B4602_RS03770, ,psos,iron chelate uptake ABC transporter family permease subunit	Present	Present	Present

Table S1. Comparative analysis of 236 transporter genes in *S. aureus* ATCC 6538, ATCC BAA-44, and ATCC 25923.

Transporter Gene No.	Gene Name	*S. aureus* ATCC 6538	*S. aureus* ATCC BAA-44	*S. aureus* ATCC 25923
85	B4602_RS03775, ,psos,ABC transporter ATP-binding protein	Present	Present	Present
86	B4602_RS03780, ,psos,siderophore ABC transporter substrate-binding protein	Present	Present	Present
87	B4602_RS04155, ,psos,amino acid transporter	Present	Present	Present
88	B4602_RS04230, ,psos,methionine ABC transporter ATP-binding protein	Present	Present	Present
89	B4602_RS04235, ,psos,ABC transporter permease	Present	Present	Present
90	B4602_RS04240, ,psos,MetQ/NlpA family ABC transporter substrate-binding protein	Present	Present	Present
91	B4602_RS04750, ,psos,ABC transporter permease	Present	Present	Present
92	B4602_RS04755, ,psos,ABC transporter permease	Present	Present	Present
93	B4602_RS04760, ,psos,ABC transporter ATP-binding protein	Present	Present	Present
94	B4602_RS04765, ,psos,ABC transporter ATP-binding protein	Present	Present	Present
95	B4602_RS04770, ,psos,peptide ABC transporter substrate-binding protein	Present	Present	Present
96	B4602_RS04780, ,psos,ABC transporter ATP-binding protein	Present	Present	Present
97	B4602_RS04785, ,psos,dipeptide ABC transporter ATP-binding protein	Present	Present	Present
98	B4602_RS04790, ,psos,ABC transporter permease	Present	Present	Present
99	B4602_RS04795, ,psos,ABC transporter permease	Present	Present	Present
100	B4602_RS04860, ,psos,magnesium transporter	Present	Present	Present
101	B4602_RS04875, ,psos,AI-2E family transporter	Present	Present	Present
102	B4602_RS04895, ,psos,MFS transporter	Present	Present	Present
103	B4602_RS05005, ,psos,ABC transporter ATP-binding protein	Present	Present	Present
104	B4602_RS05025, ,psos,ABC transporter substrate-binding protein	Present	Present	Absent
105	B4602_RS05235, ,psos,energy-coupling factor transporter transmembrane protein EcfT	Present	Present	Present

Table S1. Comparative analysis of 236 transporter genes in *S. aureus* ATCC 6538, ATCC BAA-44, and ATCC 25923.

Transporter Gene No.	Gene Name	*S. aureus* ATCC 6538	*S. aureus* ATCC BAA-44	*S. aureus* ATCC 25923
106	B4602_RS05240, ,psos,ABC transporter ATP-binding protein	Present	Present	Present
107	B4602_RS05245, ,psos,ECF transporter S component	Present	Present	Present
108	B4602_RS05375, ,psos,ABC transporter ATP-binding protein	Present	Present	Present
109	B4602_RS05380, ,psos,ABC transporter permease	Present	Present	Present
110	B4602_RS05385, ,psos,ABC transporter permease	Present	Present	Present
111	B4602_RS05390, ,psos,spermidine/putrescine ABC transporter substrate-binding protein	Present	Present	Present
112	B4602_RS05405, ,psos,divalent metal cation transporter	Present	Present	Present
113	B4602_RS05550, ,psos,heme ABC transporter substrate-binding protein IsdE	Present	Present	Present
114	B4602_RS05555, ,psos,iron ABC transporter permease	Present	Present	Present
115	B4602_RS05770, ,psos,TDT family transporter	Present	Present	Present
116	B4602_RS06410, ,psos,energy coupling factor transporter S component ThiW	Present	Present	Present
117	B4602_RS06660, ,psos,ABC transporter ATP-binding protein	Present	Present	Present
118	B4602_RS06665, ,psos,ABC transporter permease	Present	Present	Present
119	B4602_RS06820, ,psos,BCCT family transporter	Present	Present	Present
120	B4602_RS06895, ,psos,AI-2E family transporter	Present	Present	Present
121	B4602_RS06995, ,psos,ABC transporter ATP-binding protein	Present	Present	Present
122	B4602_RS07000, ,psos,ABC transporter ATP-binding protein	Present	Present	Present
123	B4602_RS07005, ,psos,ABC transporter permease	Present	Present	Present
124	B4602_RS07010, ,psos,ABC transporter permease	Present	Present	Present
125	B4602_RS07035, ,psos,phosphate ABC transporter ATP-binding protein	Present	Present	Present
126	B4602_RS07040, ,psos,phosphate ABC transporter permease PstA	Present	Present	Present
127	B4602_RS07045, ,psos,phosphate ABC transporter permease subunit PstC	Present	Present	Present

Table S1. Comparative analysis of 236 transporter genes in *S. aureus* ATCC 6538, ATCC BAA-44, and ATCC 25923.

Transporter Gene No.	Gene Name	*S. aureus* ATCC 6538	*S. aureus* ATCC BAA-44	*S. aureus* ATCC 25923
128	B4602_RS07050, ,psos,phosphate ABC transporter substrate-binding protein PstS	Present	Present	Present
129	B4602_RS07230, ,psos,PTS glucose transporter subunit IIA	Present	Present	Present
130	B4602_RS07280, ,psos,queuosine precursor transporter	Present	Present	Present
131	B4602_RS07300, ,psos,multidrug efflux MFS transporter NorB	Present	Present	Present
132	B4602_RS07555, ,psos,ECF transporter S component	Present	Present	Present
133	B4602_RS07695, ,psos,MFS transporter	Present	Present	Present
134	B4602_RS07935, ,psos,metal ABC transporter permease	Present	Present	Present
135	B4602_RS07940, ,psos,metal ABC transporter ATP-binding protein	Present	Present	Present
136	B4602_RS08170, ,psos,divalent metal cation transporter	Present	Present	Present
137	B4602_RS08730, ,psos,TSUP family transporter	Present	Present	Present
138	B4602_RS08800, ,psos,PTS transporter subunit EIIC	Present	Present	Present
139	B4602_RS08985, ,psos,MFS transporter	Present	Present	Present
140	B4602_RS09055, ,psos,arsenite efflux transporter membrane subunit ArsB	Present	Present	Present
141	B4602_RS09100, ,psos,fluoride efflux transporter CrcB	Present	Present	Present
142	B4602_RS09445, ,psos,ABC transporter permease	Present	Present	Present
143	B4602_RS09450, ,psos,ABC transporter ATP-binding protein	Present	Present	Present
144	B4602_RS09570, ,psos,amino acid ABC transporter ATP-binding protein	Present	Present	Present
145	B4602_RS09575, ,psos,ABC transporter permease subunit	Present	Present	Present
146	B4602_RS09585, ,psos,PTS transporter subunit IIC	Present	Present	Present
147	B4602_RS09770, ,psos,SAV1866 family putative multidrug efflux ABC transporter	Present	Present	Present
148	B4602_RS09800, ,psos,ABC transporter ATP-binding protein	Present	Present	Present
149	B4602_RS10145, ,psos,phenol-soluble modulin export ABC transporter permease subunit PmtD	Present	Present	Present

Table S1. Comparative analysis of 236 transporter genes in *S. aureus* ATCC 6538, ATCC BAA-44, and ATCC 25923.

Transporter Gene No.	Gene Name	*S. aureus* ATCC 6538	*S. aureus* ATCC BAA-44	*S. aureus* ATCC 25923
150	B4602_RS10150, ,psos,phenol-soluble modulin export ABC transporter ATP-binding protein PmtC	Present	Present	Present
151	B4602_RS10155, ,psos,phenol-soluble modulin export ABC transporter permease subunit PmtB	Present	Present	Present
152	B4602_RS10160, ,psos,phenol-soluble modulin export ABC transporter ATP-binding protein PmtA	Present	Present	Present
153	B4602_RS10695, ,psos,ammonium transporter	Present	Present	Present
154	B4602_RS11290, ,psos,CDF family zinc efflux transporter CzrB	Present	Present	Present
155	B4602_RS11325, ,psos,ABC transporter ATP-binding protein	Present	Present	Present
156	B4602_RS11335, ,psos,PTS mannitol transporter subunit IICB	Present	Present	Present
157	B4602_RS11440, ,psos,multidrug efflux MFS transporter LmrS	Present	Present	Present
158	B4602_RS11445, ,psos,multidrug efflux transporter SepA	Present	Present	Present
159	B4602_RS11450, ,psos,MFS transporter	Present	Present	Present
160	B4602_RS11480, ,psos,iron chelate uptake ABC transporter family permease subunit	Present	Present	Present
161	B4602_RS11485, ,psos,iron ABC transporter permease	Present	Present	Present
162	B4602_RS11490, ,psos,ABC transporter substrate-binding protein	Present	Present	Present
163	B4602_RS11510, ,psos,staphyloferrin A export MFS transporter	Present	Present	Present
164	B4602_RS11535, ,psos,BCCT family transporter	Present	Present	Present
165	B4602_RS11565, ,psos,PTS lactose/cellobiose transporter subunit IIA	Present	Present	Present
166	B4602_RS11675, ,psos,energy-coupling factor transporter transmembrane protein EcfT	Present	Present	Present
167	B4602_RS11680, ,psos,energy-coupling factor transporter ATPase	Present	Present	Present
168	B4602_RS11685, ,psos,energy-coupling factor transporter ATPase	Present	Present	Present

Table S1. Comparative analysis of 236 transporter genes in *S. aureus* ATCC 6538, ATCC BAA-44, and ATCC 25923.

Transporter Gene No.	Gene Name	*S. aureus* ATCC 6538	*S. aureus* ATCC BAA-44	*S. aureus* ATCC 25923
169	B4602_RS11870, ,psos,AEC family transporter	Present	Present	Present
170	B4602_RS11890, ,psos,efflux RND transporter permease subunit	Present	Absent	Present
171	B4602_RS11915, ,psos,MFS transporter	Present	Present	Present
172	B4602_RS11990, ,psos,molybdate ABC transporter permease subunit	Present	Present	Present
173	B4602_RS11995, ,psos,molybdate ABC transporter substrate-binding protein	Present	Present	Present
174	B4602_RS12010, ,psos,biotin transporter BioY	Present	Present	Present
175	B4602_RS12020, ,psos,ABC transporter substrate-binding protein	Present	Present	Present
176	B4602_RS12030, ,psos,urea transporter	Present	Present	Present
177	B4602_RS12325, ,psos,ABC transporter permease	Present	Present	Present
178	B4602_RS12330, ,psos,ABC transporter ATP-binding protein	Present	Present	Present
179	B4602_RS12365, ,psos,magnesium/cobalt transporter CorA	Present	Present	Present
180	B4602_RS12390, ,psos,DHA2 family efflux MFS transporter permease subunit	Present	Present	Present
181	B4602_RS12405, ,psos,multidrug effflux MFS transporter	Present	Present	Present
182	B4602_RS12435, ,psos,ABC transporter ATP-binding protein	Present	Present	Present
183	B4602_RS12440, ,psos,ABC transporter permease	Present	Present	Present
184	B4602_RS12530, ,psos,magnesium transporter CorA family protein	Present	Present	Present
185	B4602_RS12535, ,psos,PTS sucrose transporter subunit IIBC	Present	Present	Present
186	B4602_RS12560, ,psos,cation:dicarboxylase symporter family transporter	Present	Present	Present
187	B4602_RS12580, ,psos,NarK/NasA family nitrate transporter	Present	Present	Present
188	B4602_RS12645, ,psos,formate/nitrite transporter family protein	Present	Present	Present

Table S1. Comparative analysis of 236 transporter genes in *S. aureus* ATCC 6538, ATCC BAA-44, and ATCC 25923.

Transporter Gene No.	Gene Name	*S. aureus* ATCC 6538	*S. aureus* ATCC BAA-44	*S. aureus* ATCC 25923
189	B4602_RS12660, ,psos,zinc ABC transporter substrate-binding lipoprotein AdcA	Present	Present	Present
190	B4602_RS12700, ,psos,amino acid ABC transporter ATP-binding protein	Present	Present	Present
191	B4602_RS12705, ,psos,amino acid ABC transporter permease	Present	Present	Present
192	B4602_RS12710, ,psos,transporter substrate-binding domain-containing protein	Present	Present	Present
193	B4602_RS12715, ,psos,multidrug efflux MFS transporter MdeA	Present	Present	Present
194	B4602_RS12735, ,psos,cation transporter	Present	Present	Present
195	B4602_RS12775, ,psos,QueT transporter family protein	Present	Present	Present
196	B4602_RS12815, ,psos,ABC transporter ATP-binding protein	Present	Present	Present
197	B4602_RS12820, ,psos,ABC transporter ATP-binding protein	Present	Present	Present
198	B4602_RS12850, ,psos,multidrug effflux MFS transporter	Present	Present	Present
199	B4602_RS12915, ,psos,MFS transporter	Present	Present	Present
200	B4602_RS12920, ,psos,ABC transporter permease	Present	Present	Present
201	B4602_RS12925, ,psos,osmoprotectant ABC transporter substrate-binding protein	Present	Present	Present
202	B4602_RS12930, ,psos,ABC transporter permease	Present	Present	Present
203	B4602_RS12935, ,psos,ABC transporter ATP-binding protein	Present	Present	Present
204	B4602_RS12970, ,psos,iron export ABC transporter permease subunit FetB	Present	Present	Present
205	B4602_RS13015, ,psos,MFS transporter	Present	Present	Present
206	B4602_RS13020, ,psos,ABC transporter ATP-binding protein	Present	Present	Present
207	B4602_RS13025, ,psos,ABC transporter ATP-binding protein	Present	Present	Present
208	B4602_RS13030, ,psos,ABC transporter permease	Present	Present	Present
209	B4602_RS13035, ,psos,ABC transporter permease	Present	Present	Present
210	B4602_RS13040, ,psos,staphylopine-dependent metal ABC transporter substrate-binding protein CntA	Present	Present	Present

Table S1. Comparative analysis of 236 transporter genes in *S. aureus* ATCC 6538, ATCC BAA-44, and ATCC 25923.

Transporter Gene No.	Gene Name	*S. aureus* ATCC 6538	*S. aureus* ATCC BAA-44	*S. aureus* ATCC 25923
211	B4602_RS13070, ,psos,AbgT family transporter	Present	Present	Present
212	B4602_RS13155, ,psos,MFS transporter	Present	Present	Absent
213	B4602_RS13240, ,psos,MFS transporter	Present	Present	Present
214	B4602_RS13275, ,psos,ABC transporter permease	Present	Present	Present
215	B4602_RS13335, ,psos,peptide ABC transporter permease	Present	Present	Absent
216	B4602_RS13380, ,psos,PTS fructose transporter subunit IIC	Present	Present	Present
217	B4602_RS13390, ,psos,EamA family transporter	Present	Present	Present
218	B4602_RS13480, ,psos,fatty acid efflux MMPL transporter FarE	Present	Present	Present
219	B4602_RS13575, ,psos,PTS transporter subunit IIC	Present	Present	Present
220	B4602_RS13645, ,psos,ferrous iron transporter B	Present	Present	Present
221	B4602_RS13830, ,psos,BCCT family transporter	Present	Present	Present
222	B4602_RS13855, ,psos,CitMHS family transporter	Present	Present	Present
223	B4602_RS13900, ,psos,ABC transporter permease	Present	Present	Present
224	B4602_RS13905, ,psos,ABC transporter ATP-binding protein	Present	Present	Present
225	B4602_RS14005, ,psos,PTS transporter subunit EIIA	Present	Present	Present
226	B4602_RS14225, ,psos,energy-coupling factor transporter transmembrane protein EcfT	Present	Present	Present
227	B4602_RS14230, ,psos,ABC transporter ATP-binding protein	Present	Present	Present
228	B4602_RS14235, ,psos,ECF-type riboflavin transporter substrate-binding protein	Present	Present	Present
229	B4602_RS14275, ,psos,EamA family transporter RarD	Present	Present	Present
230	B4602_RS14285, ,psos,HoxN/HupN/NixA family nickel/cobalt transporter	Present	Present	Present
231	B4602_RS14300, ,psos,peptide resistance ABC transporter ATP-binding subunit VraD	Present	Present	Present
232	B4602_RS14305, ,psos,peptide resistance ABC transporter permease subunit VraE	Present	Present	Present

Table S1. Comparative analysis of 236 transporter genes in *S. aureus* ATCC 6538, ATCC BAA-44, and ATCC 25923.

Transporter Gene No.	Gene Name	*S. aureus* ATCC 6538	*S. aureus* ATCC BAA-44	*S. aureus* ATCC 25923
233	B4602_RS14310, ,psos,peptide resistance ABC transporter activity modulator VraH	Present	Present	Present
234	B4602_RS10590, ,psos,iron-hydroxamate ABC transporter substrate-binding protein	Present	Absent	Absent
235	B4602_RS13255, ,psos,MFS transporter	Present	Absent	Absent
236	B4602_RS14330, ,psos,peptide resistance ABC transporter activity modulator VraH	Present	Absent	Absent

Table S2. Primer sequence of target genes *femX*, *femA* and *femB* and reference gene 16S rRNA.

Genes	Primer Sequence
femX	Forward- 5’-GCCATGGAAAAGATGCATATCAC-3’<br>Reverse- 5’- CTCGAGTTTTCGTTTTAATTTACGAG-3’
femA	Forward- 5’-AGA CAA ATA GGA GTA ATG AT-3’<br>Reverse- 5’-AAA TCT AAC ACT GAG TGA TA-3’
femB	Forward- 5’-TTA CAG AGT TAA CTG TTA CC-3’<br>Reverse- 5’-ATA CAA ATC CAG CAC GCT CT-3’
16S rRNA	Forward- 5’-AGA GTT TGA TCC TGG CTC AG-3′<br>Reverse- 5’- TAC GGC TAC CTT GTT ACG ACT T-3’

Table S3. The binding affinity of BCA and catechin gallate against *femA and femB* and their interacting key amino acid residues.

Genes	Protein Code	Ligand	Binding affinity (kcal/ mol)	Interacting amino acid residues	Type of interaction
femX	6SNR	BCA	-7.1	TYR320	Hydrogen bond
				GLY355, GLN143, TYR212, LYS33, LEU23, ASP22, TYR317, ASP353, LYS384, THR147	Van der Waals
				ARG74	Pi-Cation
				LEU24, PRO144, LEU319, TRP29	Pi-Alkyl
		Catechin gallate	-8.4	HIS334, ASN333, ARG202, PHE373	Hydrogen bond
				ASN167, TRP377, LEU331, ARG328, GLN337, MET336, TYR318, GLY321, ALA322, SER305, THR199, ALA372, VAL376	Van der Waals
				SER 323	Unfavorable Acceptor-Acceptor
		Rutin	-8.6	LEU137, SER138, ARG74, ASP22, LYS33, TYR320, LYS374, GLY355	Hydrogen bond
				LYS139, ILE142, THR147, ASP353, GLN143, LEU319, TYR212, TRP29, PHE354, LEU370, GLY356, PRO144, HIS367	Van der Waals
				TYR317, LEU24	Pi-alkyl
femA	1LRZ	BCA	-7.7	LEU153, ILE155, TYR327, GLY365, and LYS383	Hydrogen bond
				PHE149, GLN154, TYR328, ALA329, GLY330, PHE363, TYR364	Van der Waals
		Catechin gallate	-9.1	GLY365, LYS383, GLN154, and ILE155	Hydrogen bond
				ALA329	Pi-Alkyl
				PHE149, TYR327	Pi-pi

Table S3. The binding affinity of BCA and catechin gallate against *femA and femB* and their interacting key amino acid residues.

Genes	Protein Code	Ligand	Binding affinity (kcal/ mol)	Interacting amino acid residues	Type of interaction
				LEU153	Unfavorable Acceptor-acceptor
				PHE363, TYR364, TYR328, GLY330, MET208, GLU375, ASP376, ASP150, VAL152	Van der Waals
		Rutin	-8.7S	ARG228, LYS33, THR59, GLN154, and VAL152	Hydrogen bond
				THR24, PHE23, ASN73, TYR71, LEU32, VAL61, TYR69, GLU36, ASN227, TYR38, PHE224	Van der Waals
				PRO151, HIS29	Unfavorable Acceptor-Acceptor
femB	Q2FYR1	BCA	-7.5	LYS381, GLY363, VAL377, PHE380	Hydrogen bond
				PHE380, PRO340, VAL377	Pi-pi
				PHE218, TYR150, VAL156, SER330, TYR325, PHE361, PHE326, HIS344, TYR362	Van der Waals
		Catechin gallate	-9.3	PHE326, GLY328	Hydrogen bond
				TYR150, PHE218	Pi-pi
				GLU373, ASP374, VAL377, LYS221, SER220, SER327, TYR362, TYR325, THR212, GLY329, TYR209, THR152, ASP151, SER154, VAL156	Van der Waals
		Rutin	-9.9	VAL156, SER154, GLY328, SER220, TYR362, GLY363, TYR325	Hydrogen bond
				PHE128	Pi-pi
				VAL377, ALA216, THR212, GLU213, TYR209, GLU373, SER327, TYR150, PHE326, LYS381, PHE361, LYS221, GLN155, ASP151	Van der Waals

Table S3. The binding affinity of BCA and catechin gallate against *femA and femB* and their interacting key amino acid residues.

**Genes**	**Protein Code**	**Ligand**	**Binding affinity (kcal/ mol)**	**Interacting amino acid residues**	**Type of interaction**

Table S4. Pharmacokinetics of BCA compared to flavonoids predicted to have a binding interaction against *femA* and *femB.*

**Property**	**Model**	**BCA**	**Catechin gallate**	**Rutin**
Absorption	Water solubility (log mol/L)	-3.735	-2.911	-2.892
	Caco2 permeability (log Papp in 10-6 cm/s)	0.897	-1.264	-0.949
	Intestinal absorption (human)	93.028	62.096	23.446
	Skin Permeability (% Absorbed)	-2.737	-2.735	-2.735
	P-glycoprotein substrate (log Kp)	Yes	Yes	Yes
	P-glycoprotein I inhibitor	No	No	No
	P-glycoprotein II inhibitor	No	Yes	No
Distribution	VDss (human)	-0.341	0.664	1.663
	Fraction unbound (human) (Fu)	0.03	0.158	0.187
	BBB permeability (log BB)	-0.221	-1.847	-1.899
	CNS permeability (log PS)	-2.115	-3.743	-5.178
Metabolism	CYP2D6 substrate	No	No	No
	CYP3A4 substrate	Yes	No	No
	CYP1A2 inhibitor	Yes	No	No
	CYP2C19 inhibitor	Yes	No	No
	CYP2C9 inhibitor	Yes	No	No
	CYP2D6 inhibitor	No	No	No
	CYP3A4 inhibitor	No	No	No
Excretion	Total Clearance (log ml/min/kg)	0.247	-0.169	-0.369

Table S3. The binding affinity of BCA and catechin gallate against *femA and femB* and their interacting key amino acid residues.

Genes	Protein Code	Ligand	Binding affinity (kcal/ mol)	Interacting amino acid residues	Type of interaction

	Renal OCT2 substrate	No	No	No
Toxicity	AMES toxicity	No	No	No
	Max. tolerated dose (human) (log mg/kg/day)	0.4	0.449	0.452
	hERG I inhibitor	No	No	No
	hERG II inhibitor	No	Yes	Yes
	Oral Rat Acute Toxicity (LD50) (mol/kg)	1.851	2.558	2.491
	Oral Rat Chronic Toxicity (LOAEL) (log mg/kg_bw/day)	1.142	2.777	3.673
	Hepatotoxicity	No	No	No
	Skin Sensitization	No	No	No
	T.Pyriformis toxicity (log ug/L)	0.515	0.285	0.285
	Minnow toxicity (log mM)	0.681	6.146	7.677

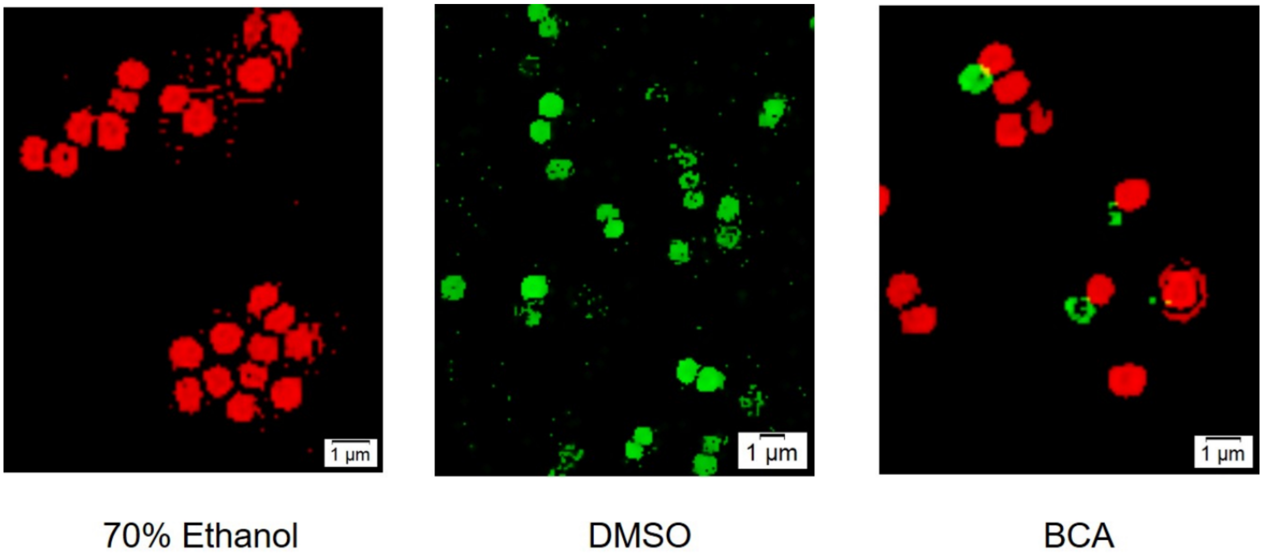

Figure S1. Fluorescent microscopy at 200X zoom of MDRSA treated with 70% Ethanol, DMSO, and BCA.

.

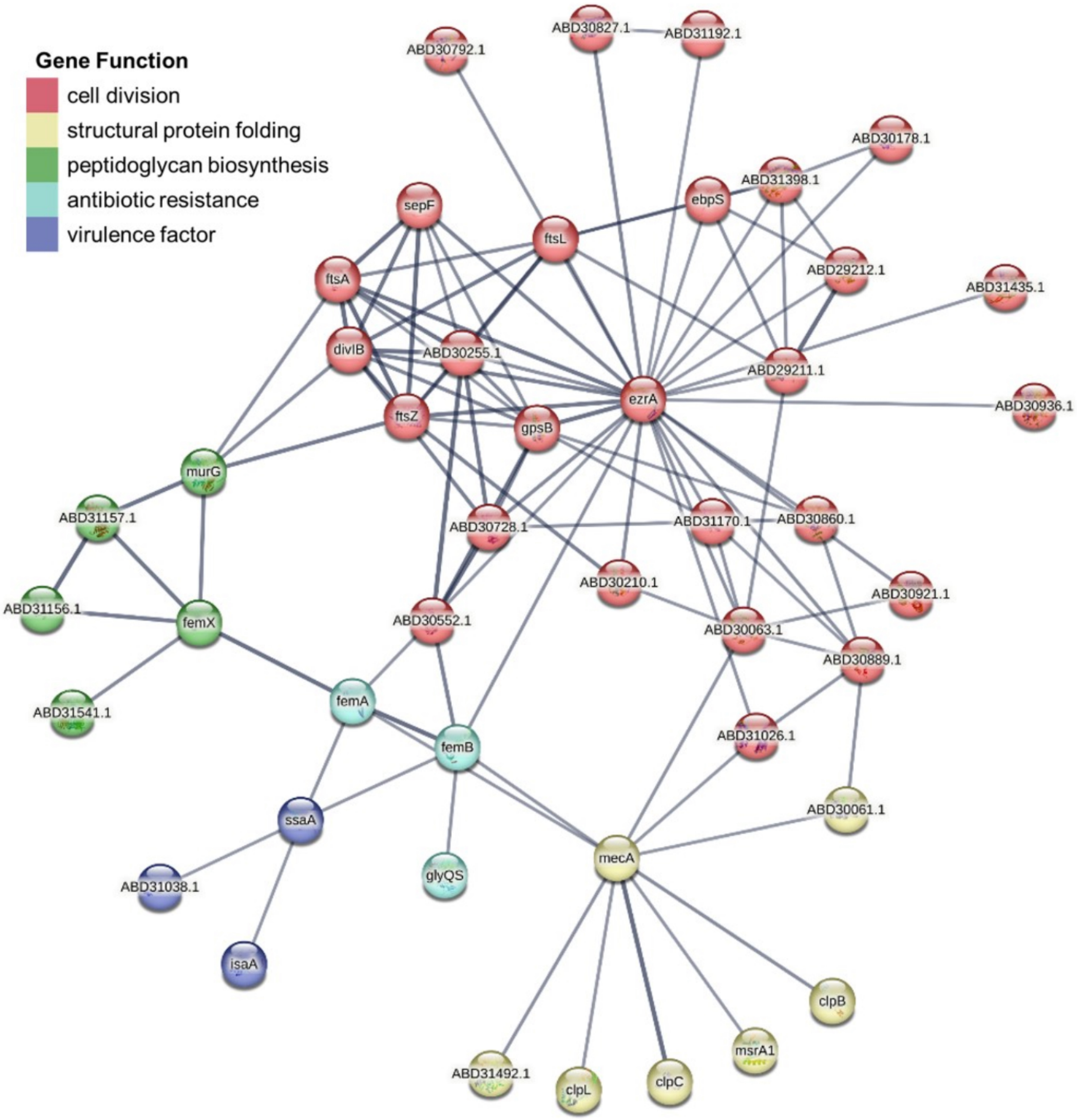

Figure S2. Gene enrichment analyses of *femA* and *femB* showed its association to other genes that play a key role in other biosynthesis pathway.
